# Pseudo Natural Products—Chemical Evolution of Natural Product Structure

**DOI:** 10.1002/anie.202016575

**Published:** 2021-03-23

**Authors:** George Karageorgis, Daniel J. Foley, Luca Laraia, Susanne Brakmann, Herbert Waldmann

**Affiliations:** ^1^ Max-Planck Institute of Molecular Physiology Otto-Hahn Strasse 11 44227 Dortmund Germany; ^2^ Current address: School of Physical and Chemical Sciences University of Canterbury Private Bag 4800 Christchurch 8140 New Zealand; ^3^ Current address: Department of Chemistry Technical University of Denmark, kemitorvet 207 2800 Kgs. Lyngby Denmark; ^4^ Faculty of Chemistry and Chemical Biology TU Dortmund University Otto-Hahn Strasse 4a 44227 Dortmund Germany

**Keywords:** biological activity, chemical biology, fragment-based design, natural products, natural selection

## Abstract

Pseudo‐natural products (PNPs) combine natural product (NP) fragments in novel arrangements not accessible by current biosynthesis pathways. As such they can be regarded as non‐biogenic fusions of NP‐derived fragments. They inherit key biological characteristics of the guiding natural product, such as chemical and physiological properties, yet define small molecule chemotypes with unprecedented or unexpected bioactivity. We iterate the design principles underpinning PNP scaffolds and highlight their syntheses and biological investigations. We provide a cheminformatic analysis of PNP collections assessing their molecular properties and shape diversity. We propose and discuss how the iterative analysis of NP structure, design, synthesis, and biological evaluation of PNPs can be regarded as a human‐driven branch of the evolution of natural products, that is, a chemical evolution of natural product structure.

## Graphical abstract

Pseudo‐natural products provide new opportunities in the discovery of bioactive small molecules and can be regarded as a human‐driven chemical evolution of natural product structure.

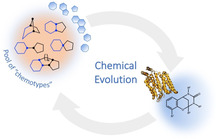

## 1 Introduction

Small molecules are powerful tools for the dissection of complex biological processes due to their ability to acutely modulate their biological targets in a tuneable manner, and are the dominant chemical entities^[1]^ in our arsenal to treat disease.^[2]^ The discovery of novel small molecules with suitable properties to interrogate biological phenomena in a time‐resolved manner is underpinned by the ability to design and prepare new molecular scaffolds. However, the vastness of chemical space^[3]^ renders its complete exploration by means of synthesis impossible, hampering our ability to efficiently discover new bioactive molecular scaffolds.^[4]^

**Figure 1:**
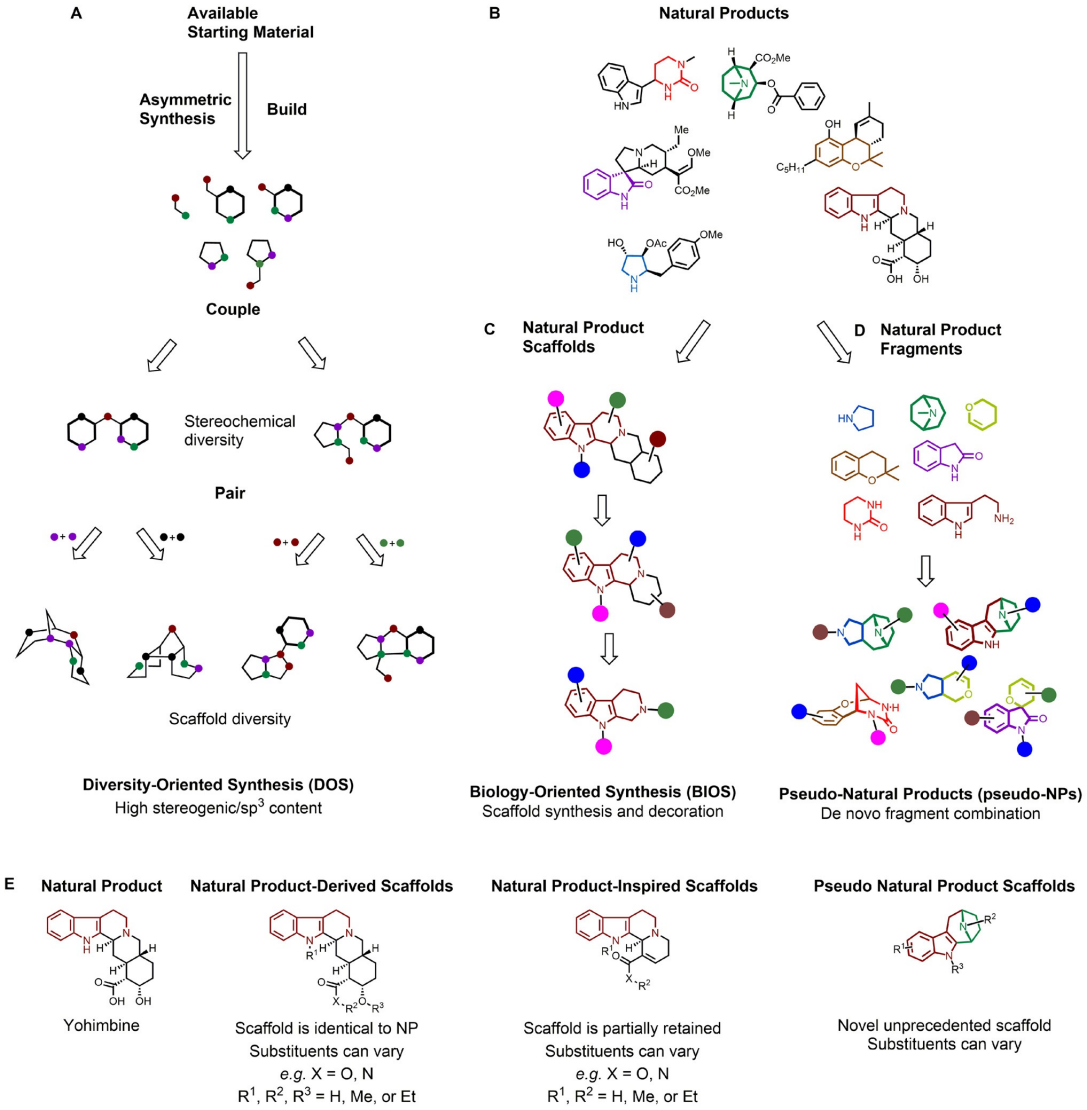
Approaches for the design and preparation of novel biologically relevant molecular scaffolds. A) DOS employs a build‐couple‐pair approach leading to diverse scaffolds that can be considered NP‐like. B) Natural products are secondary metabolites that provide inspiration for the discovery of bioactive small molecules. C) BIOS draws inspiration from NPs, preparing analogues of NP‐derived scaffolds with reduced structural complexity. D) Pseudo‐natural products emerge from unprecedented combinations of NP‐derived fragments. E) Differences between (left to right) NPs, NP‐derived scaffolds (i.e. scaffold is identical to NP scaffold), NP‐inspired scaffolds (i.e. scaffold is closely related to NP‐scaffold^[13]^), and pseudo‐NP scaffolds.

To address these challenges, several complementary approaches have been developed. For example, diversity‐oriented synthesis (DOS)^[5]^ is an approach aimed towards the preparation of compound libraries whereby creation of molecular diversity is an embedded part of the synthetic strategy (Figure 1 A). DOS exploits intermolecular building‐block coupling, followed by intramolecular functional group pairings[^6^, ^7^] resulting in molecular scaffolds with high stereochemical content and fractions of sp^3^‐hybridised centres. DOS has led to the discovery of a range of bioactive small molecules,^[8]^ which have inspired drug discovery programmes^[9]^ or have been used as tools to investigate biological processes.^[10]^

Natural products (NPs) are a rich and continuously explored resource in the search for bioactive small molecules.^[11]^ Biology‐oriented synthesis (BIOS) takes inspiration from NP structures to guide the synthesis of biologically relevant compound collections. BIOS employs a hierarchical classification of NP scaffolds, generated by a computational algorithm,^[12]^ to select simplified, NP‐derived and ‐inspired scaffolds which retain their ability to modulate biological systems (Figure 1 B,C,E)^[13]^ yet are more synthetically tractable. BIOS exploits the gaps in chemical space not covered by NPs and facilitates the preparation of derivatives. However, the partial retention of the guiding NP‐scaffold limits the chemical space that can be explored. Additionally, BIOS scaffolds may also inherit the same kind of bioactivity as the guiding NPs, thus limiting the exploration of biological space.^[14]^

To overcome these limitations and take advantage of the biological relevance of NPs, the design of novel scaffolds can benefit from the efficient sampling of chemical space offered by fragment‐based compound design.^[15]^ This argument is supported by the fact that NPs may already be fragment‐sized,^[16]^ or can be converted into fragment‐sized ring‐systems,^[17]^ and the properties of NPs are retained in these NP‐derived fragments.^[18]^ Thus, combining NP‐derived fragments in unprecedented ways can provide access to molecular scaffolds which inherit the biological characteristics of NPs, yet lie in biologically relevant regions of chemical space not attainable by nature. We have termed these compounds “pseudo‐natural products” (pseudo‐NPs), as these novel scaffolds would not be accessible through current naturally occurring biosynthetic pathways and can be regarded as non‐biogenic fusions of NP‐derived fragments (Figure 1 B,D).[^19^, ^20^] The term “pseudo‐natural product” has previously been used sparingly, for example, to describe cyclic peptides,[^21^, ^22^] and products of altered or intercepted biosynthetic pathways.[^23^, ^24^] We have demonstrated how alternate scaffold connectivity patterns can be used to design new pseudo‐NP scaffolds which occupy different regions of chemical space.^[20]^ This design process can be thought as a chemical counter‐part to naturally occurring (biologically‐driven) evolution of compound structure, which relies on a simple optimisation algorithm comprising diversification and selection, as introduced below.

In this Minireview we describe the development of the pseudo‐NP concept, the principles of pseudo‐NP‐library design, and their relationship to the biological evolution of NP structure. We describe syntheses of pseudo‐NP compound collections and their investigation in different biological settings, showing that pseudo‐NPs can harbour novel bioactivity not shared by the guiding NPs.

## 2 Design Principles for Pseudo‐Natural Products

In general, new pseudo‐NP scaffolds preferably have a high degree of three‐dimensional character, as chirality and stereogenic content contribute to biological relevance and bioactivity.[^25^, ^26^, ^27^] To provide structurally distinct pseudo‐NPs, NP‐derived fragments with complementary heteroatom content may be combined (e.g. N and O). Combination of fragments sourced from different organisms or biosynthetic pathways may increase the novelty of the resulting scaffold, and its biological relevance, by combining features from discrete and otherwise unrelated areas of chemical space.

**Figure 2:**
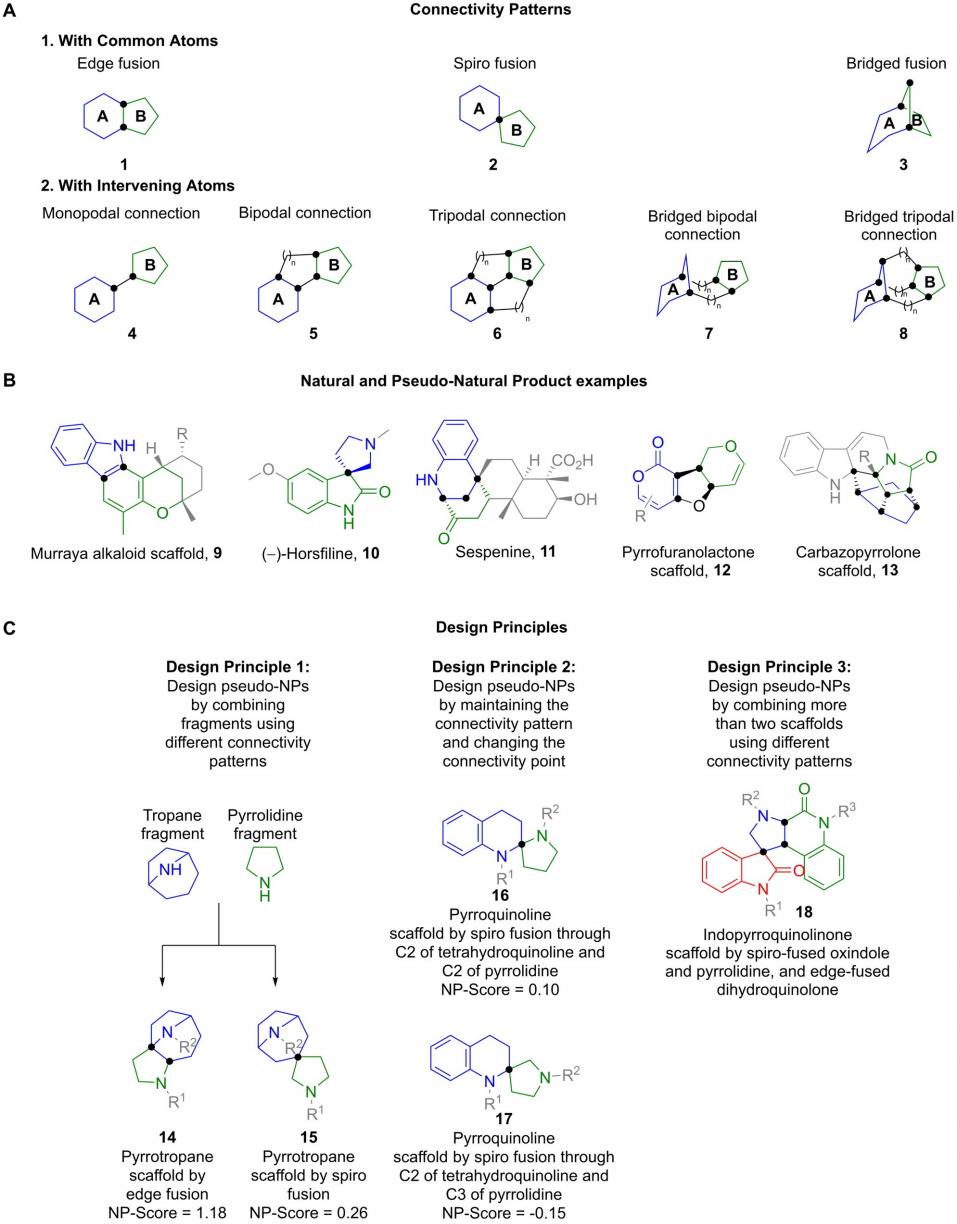
Design principles for pseudo‐NP scaffolds. In general, pseudo‐NP scaffolds should have high stereogenic content and three‐dimensional character, complementary heteroatom content, and combine fragments from different sources. Parts of structures have been greyed for clarity. Fragments are coloured for distinction. Black dots represent connectivity atoms. A) Examples of connectivity patterns illustrated with abstract structures. B) Examples of the connectivity patterns in natural and pseudo‐natural product scaffolds. C) Design principles for pseudo‐NPs. Pseudo‐NP scaffolds arise by combining different fragments using different connectivity patterns, or by combining the same fragments and the same connectivity patterns through different common atoms. It is also possible to combine more than two fragments.

NP‐derived fragments can be connected in entirely novel arrangements not found in nature (see below), or, to retain certain biologically relevant components in the resulting pseudo‐NP, through specific structural patterns already encountered in NPs. These connectivity patterns can be classed into two sets; those where the connected fragments share common atoms (Figure 2, Panel A, **1**–**3**), and those where the fragments are connected through intervening atoms (Figure 2, Panel A, **4**–**8**). For example, two fragments can be combined through a common edge and sharing two common atoms as in the abstract scaffold **1**. This connectivity pattern can be observed in alkaloids containing an indole and a chromane fragment, such as **9** ^[28]^ (Figure 2, Panel B). Alternatively, fragments can be connected through a single common atom resulting in a spiro‐fusion (Figure 2, Panel A, **2**), as in the case of the NP (−)‐horsfiline, **10**.^[29]^ If two fragments are connected through three consecutive common atoms, such a pattern would result in a bridged‐fusion (Figure 2, Panel A, **3**). An example of this connectivity pattern can be seen in the NP sespenine, **11**.^[30]^ The bipodal connection can be observed in pseudo‐natural product scaffold **12**,^[31]^ and an example of a bridged tripodal connection is found in structures such as scaffold **13**.^[32]^

Exploiting different connectivity patterns to connect NP‐fragments gives rise to pseudo‐NP scaffolds which can be used to probe distinct regions of biologically relevant chemical space (Figure 2, Panel C, Design Principle 1).^[20]^ For example, scaffolds **14** and **15** are pyrrotropanes stemming from the use of two different connectivity patterns; an edge‐fusion and a spiro‐fusion, respectively. In addition, combinations of the same NP‐fragments, using the same connectivity pattern, can also result in further regioisomeric pseudo‐NP scaffolds by changing the connectivity points between the connecting fragments (Figure 2, Panel C, Design Principle 2). An example can be seen when comparing pyrroquinolines **16** and **17**. Additionally, these connectivity patterns have been identified, and can be exploited for the combination of more than two NP‐derived fragments at a time (Figure 2, Panel C, Design Principle 3). Taken together, these design principles can be used to reveal unexplored areas of chemical space with potentially high bioactivity value.

## 3 Synthesis of Pseudo‐NP Libraries

### 3.1 Chemical Synthesis of Pseudo‐NPs by NP Fragment Fusion

A significant strategic approach for the preparation of pseudo‐natural product libraries is to develop and apply novel synthetic chemistries that enable the de novo fusion of ring systems from natural products in fusion patterns unprecedented in nature, ideally combining ring systems that are normally not found together.^[20]^ Here we provide some examples of pseudo‐NPs that meet the design criteria described in the section above.

**Scheme 1:**
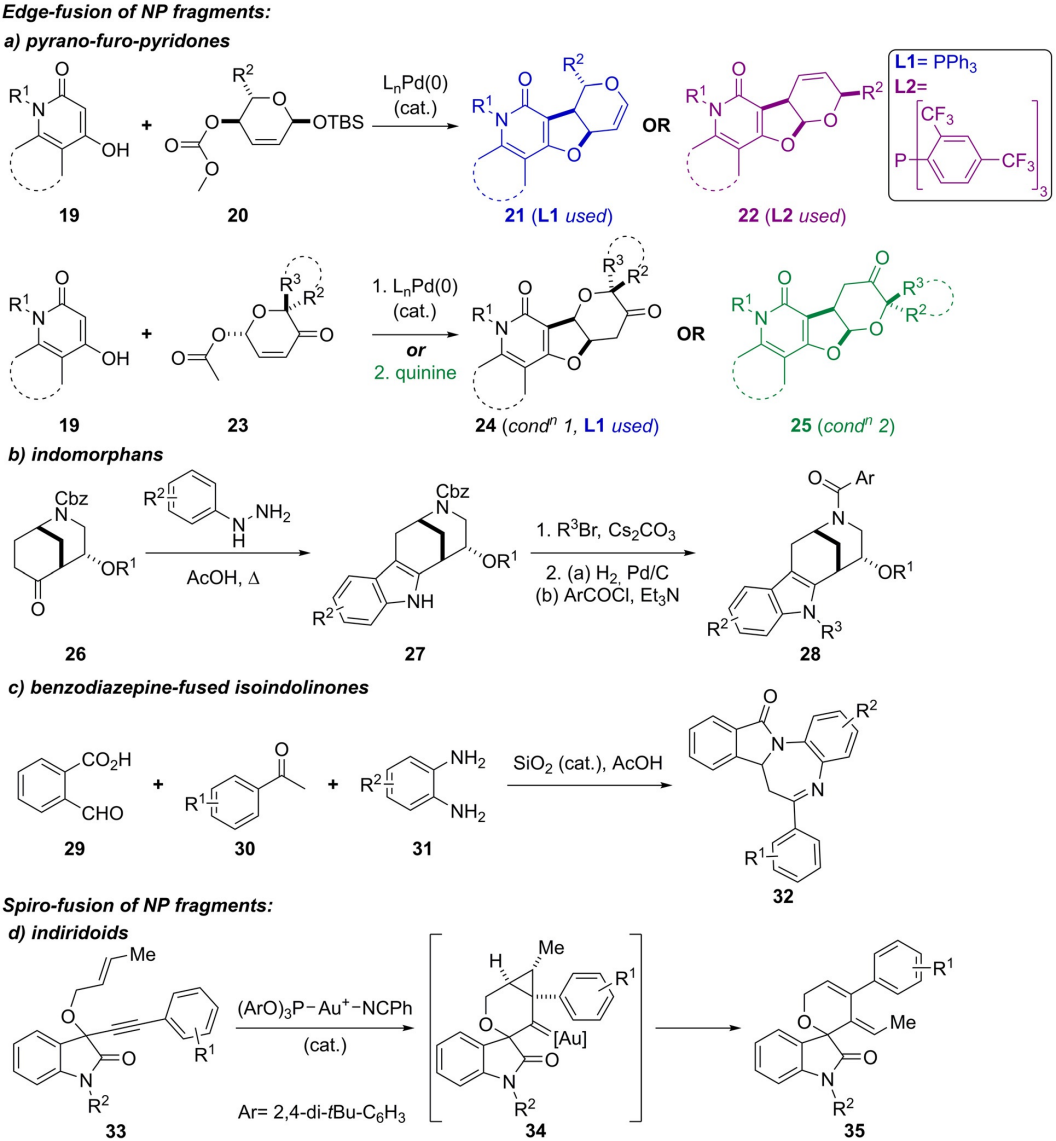
De novo synthesis of edge‐ and spiro‐fused pseudo‐NPs. a–c) Synthesis of edge‐fused pseudo‐NPs, including a) pyrano‐furo‐pyridones **21**–**22** and **24**–**25**, by Pd‐ or quinine‐catalysed cascades; b) indomorphans **26**, by Fischer indolisations; c) isoindolinones **32**, by a mesoporous silica nanoparticle‐catalysed multi‐component reaction. d) Synthesis of spiro‐fused indiridoids **35** by an Au^I^‐catalysed cascade.

*Edge‐fusion of NP fragments*: In work from our group, pyrano‐furo‐pyridones **21**–**22** and **24**–**25**, combining 2‐pyridone and (dihydro)pyran fragments, were prepared by annulation reactions (Scheme 1 a).^[33]^ These included Pd‐catalysed Tsuji–Trost cascades (→**21**–**22**), Pd‐catalysed Tsuji–Trost oxa‐Michael cascades (→**24**), and quinine‐mediated Michael transacetalisation cascades (→**25**). Subsequent modifications provided a library of >160 compounds. Indomorphans **28**, combining indole‐ and morphan‐alkaloid fragments, were prepared from known bicyclic ketones **26**, which were subjected to Fischer indolisations.^[34]^ The resulting compounds were decorated to provide a screening collection of >40 compounds (Scheme 1 b). Yu and Liu described the synthesis of benzodiazepine‐fused isoindolinones **32**, using a mesoporous silica nanoparticle‐catalysed multi‐component reaction (Scheme 1 c).^[35]^

*Spiro‐fusion of NP fragments*: The spirocyclic indiridoids **35**, combining characteristic substructures of iridoid terpenes, oxindole, and dihydropyran fragments, were prepared using an Au^I^‐catalysed reaction cascade involving a 6‐*endo*‐*dig* ene–yne cyclisation followed by ring opening and rearrangement (Scheme 1 d). Variation of the Au catalyst and the substitution pattern on the starting material gave rise to differentially fused scaffolds (not shown).^[36]^

**Scheme 2:**
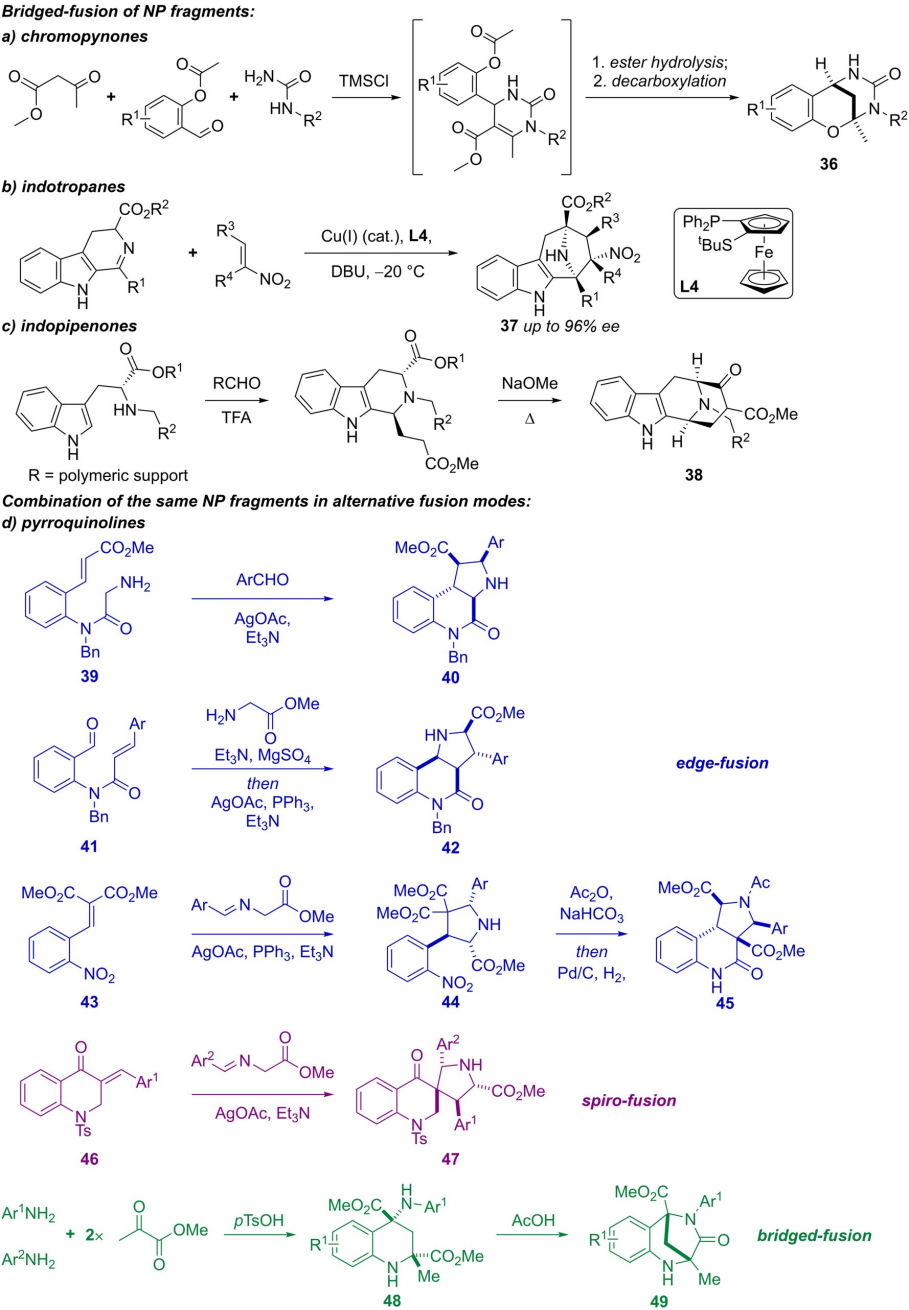
De novo synthesis of bridged‐fused pseudo‐natural products and combination of pyrrolidine and tetrahydroquinoline NP fragments in alternative fusion modes. a–c) Synthesis of bridge‐fused pseudo‐NPs, including a) chromopynones **36**, via a multicomponent reaction; b) indotropanes **37**, using a a Cu^I^‐catalysed enantioselective 1,3‐dipolar cycloaddition; c) indopipenones **38**, via an enantioselective Pictet–Spengler reaction. d) Combination of the same NP fragments in different connectivity patterns, using Ag‐catalysed 1,3‐dipolar cycloadditions as a general unifying approach. Bridged bicycles **49** were prepared via a Povarov‐type reaction.

*Bridged‐fusion of NP fragments*: Chromane and tetrahydropyrimidinone fragments were combined to give chromopynones **36**, which were synthesised in a one‐pot procedure involving a Biginelli reaction (Scheme 2 a). Indotropanes **37** combined tropane and indole fragments by harnessing a Cu^I^‐catalysed enantioselective intermolecular 1,3‐dipolar cycloaddition (Scheme 2 b). Indoles were also fused with piperidones to prepare indopipenones **38**, using a one‐pot process that included the use of an enantioselective Pictet–Spengler reaction (Scheme 2 c).^[37]^

*Combination of the same NP fragments using different connectivity patterns* (Scheme 2 d): A 155‐membered pyrroquinoline pseudo‐NP collection was generated by combining the tetrahydroquinoline and pyrrolidine fragments in eight different molecular connectivity/regioisomeric arrangements.^[38]^ Notably, scaffolds **40**, **42**, and **45** have the same fused scaffold at the graph level,^[39]^ but vary the position of the pyrrolidine nitrogen, whilst **47** has spirocyclic connectivity, and **49** merges the scaffolds in a bridged fashion. A unifying synthetic approach harnessing Ag‐catalysed 1,3‐dipolar cycloadditions of azomethine ylides with electron deficient alkenes delivered the majority of the pyrroquinoline scaffolds (**40**, **42**, and **45**). Bridged bicyclic compounds **49** were prepared via Povarov‐type dimerisation of enamines, generated from anilines and methyl pyruvate (→**48**), followed by acid‐mediated lactamisation. Oxidised versions of scaffolds **40**, **42**, and **45**, in which the pyrrolidine ring was aromatised to the corresponding pyrrole were also prepared, either by direct oxidation of **40** or **42** with DDQ, or, to give the oxidised versions of scaffold **45**, by developing a novel reaction in which azomethine ylides were reacted with quinolinium salts (not shown).

### 3.2 Pseudo‐NPs from Existing NPs

**Scheme 3:**
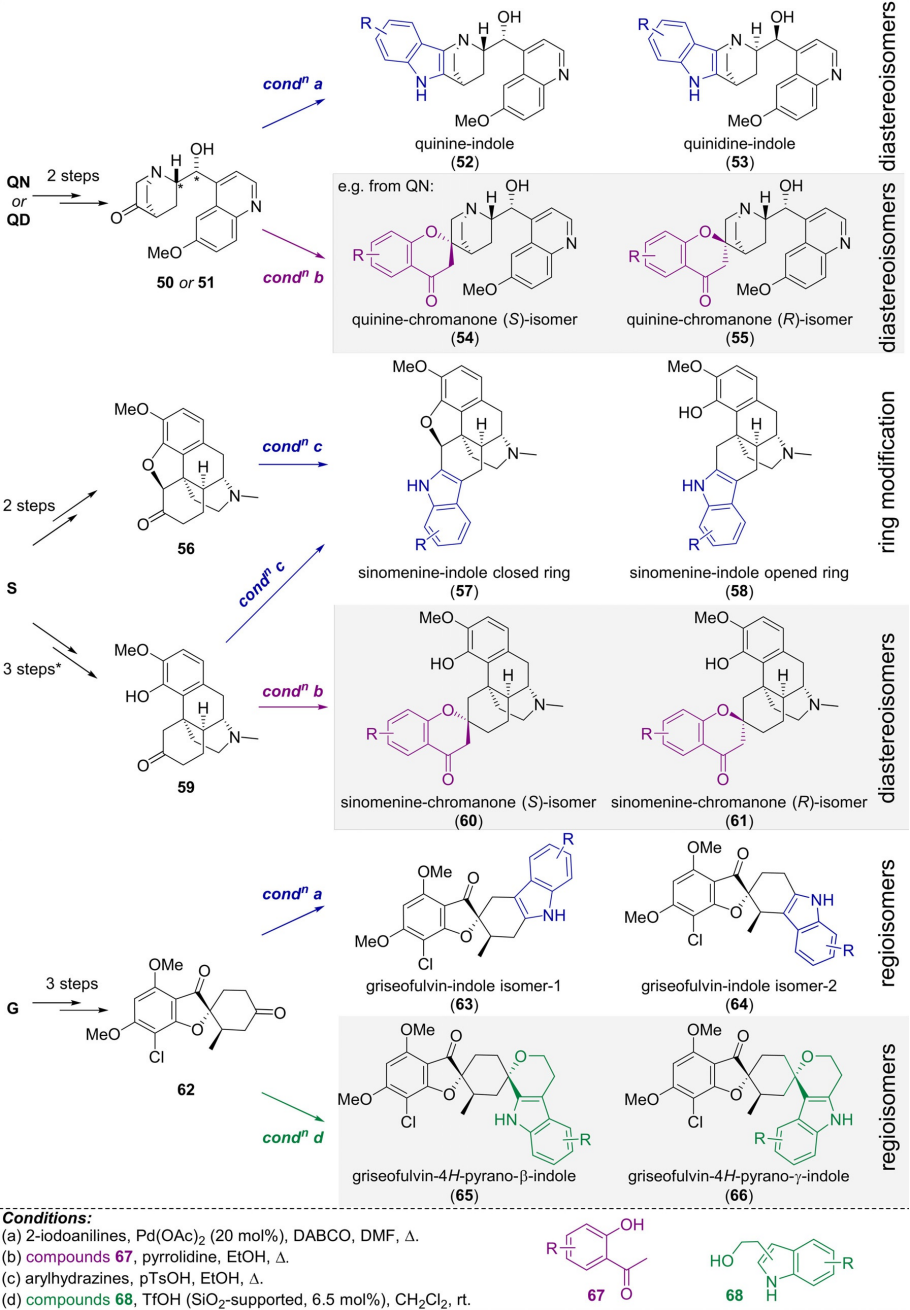
Synthesis of 244 pseudo‐NPs from natural product fragments using indolisation reactions (blue arrows, conditions a and c); oxa‐Pictet–Spengler reactions (green arrows, condition d); and Kabbe reactions (pink arrows, condition c). * 1 step from **56**.

Natural products themselves can be fragment‐sized^[16]^ and can therefore serve as starting points for new pseudo‐NPs (Scheme 3).[^40^, ^41^] This design principle was employed in unprecedented fusions of the highly NP‐prevalent indole or chromanone ring systems with readily accessible NP‐fragments derived from commercially available Cinchona alkaloids quinine (**QN**) and quinidine (**QD**), griseofulvin (**G**), and sinomenine (**S**) (structures not shown).[^40^, ^41^] Firstly, ketone fragments (**50**, **51**, **56**, **59**, and **62**) were derived from the natural products in short synthetic sequences (≤3 steps). The ketones were harnessed in a range of annulation reactions, including edge‐fusion by indolisations (blue arrows; compounds **52**–**53**, **57**–**58**, and **63**–**64**), and spiro‐fusions by either oxa‐Pictet–Spengler reactions (green arrow; compounds **65** and **66**), or Kabbe condensations (pink arrows; compounds **54**–**55** and **60**–**61**). Indolisation was achieved either by Pd‐catalysed Heck‐type annulation (conditions a) or Fischer indolisation (conditions c). The indolisations produced separable regioisomers from ketone **62** (→**63** and **64**). Compounds derived from the Kabbe condensation in each case provided two separable diastereomers at the spirocyclic point of fragment connection (e.g. **54**, **55** and **60**, **61**). Overall, a library of 244 compounds was prepared.

**Scheme 4:**
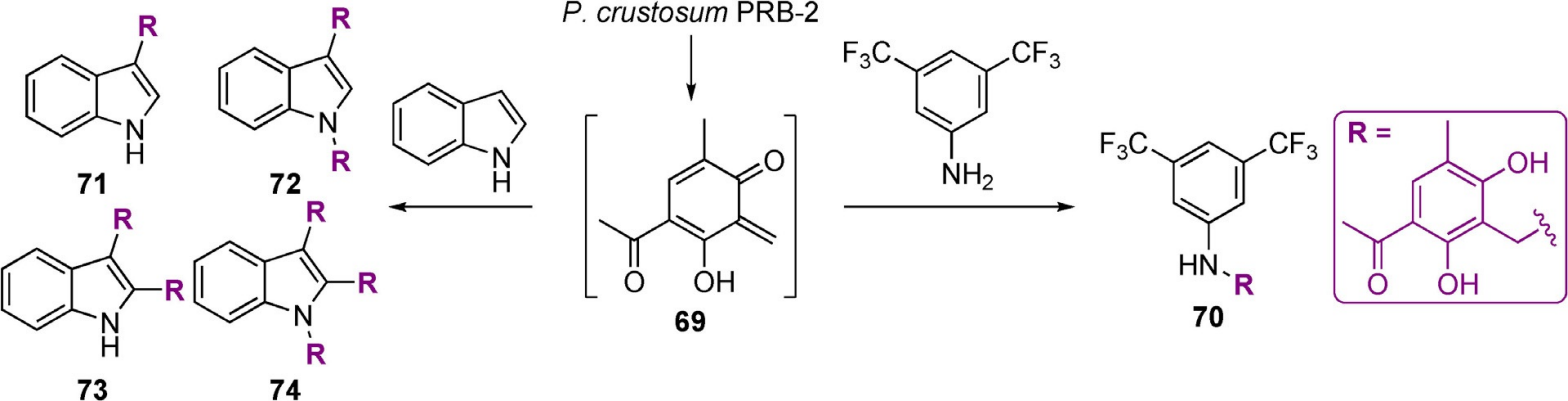
Synthesis of pseudo‐natural products by incubation of nucleophiles with *P. crustosum* PRB‐2. Exploiting a microorganism to source the reactive *ortho*‐quinone methide **69** provided access to a total of 15 diverse compounds including chemotypes **70**–**74**.

*Synthesis of pseudo‐NPs using microorganisms*: Li described the synthesis of novel pseudo‐natural products from an *ortho*‐quinone methide **69**, produced by *P. crustosum* PRB‐2, a clavatol‐producing fungus. *P. crustosum* PRB‐2 was directly incubated with alternate indole‐ and aniline nucleophiles, which reacted with the *ortho*‐quinone methide **69**, a Michael acceptor, to produce a total of 15 compounds including **70**–**74** (Scheme 4).^[42]^

## 4 Cheminformatic Analysis

**Figure 3:**
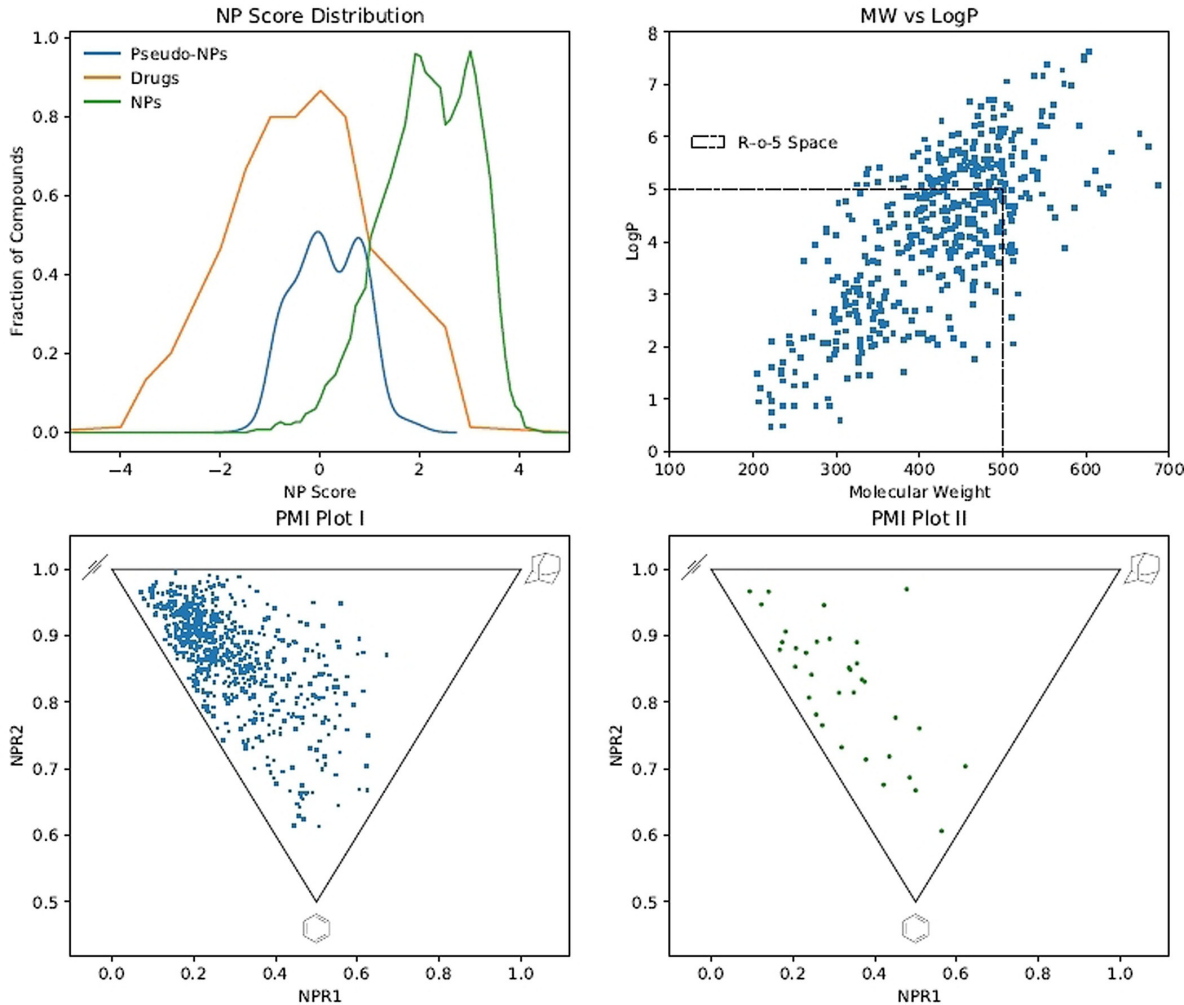
Cheminformatic analyses of pseudo‐NPs. Top left: NP‐score distributions of pseudo‐NPs (blue line), approved and experimental drugs in DrugBank, and NPs in the ChEMBL repository. Top right: Plot of molecular weight against lipophilicity of each molecule: 76 % of compounds fall within the “rule‐of‐five” space denoted by the dashed black line. Bottom left: PMI plot demonstrating the high degree of three‐dimensional character of pseudo‐NPs, as most of the molecules lie away from the rod‐disc‐like axis. Bottom right: PMI plot of selected natural products and non‐naturally occurring bioactive compounds, demonstrating a similar breadth and distribution with pseudo‐NPs (see SI, Scheme S7 for chemical structures).

To examine the relationship between pseudo‐NPs and NPs the natural product likeness score (NP‐Score)^[43]^ was calculated for selected pseudo‐NP classes reported by us so far (see SI, Figures S1–S6). Briefly, this cheminformatic tool compares connection pathways between atoms (up to six) found in NPs and a reference set of synthetic molecules. For comparison, we also determined the NP‐Score of the set of experimental and approved drugs in DrugBank^[44]^ (Figure 3, top left, orange) and the set of NPs in the ChEMBL repository^[45]^ (Figure 3, top left, green). Most of the ChEMBL NPs display a NP‐Score between 0 and 4, while the molecules in DrugBank display a much wider NP‐Score distribution between −4 and 3. There is significant overlap between these two sets, as a range of molecules in the DrugBank set are either inspired by NPs or are NPs themselves.[^46^, ^47^, ^48^] Pseudo‐NPs (Figure 3, top left, blue) however, display a narrower NP‐Score distribution between −2 and 2.^[20]^ This observation may appear counterintuitive at first glance, as pseudo‐NPs consist of NP‐derived fragments or fragment‐sized NPs. The lower collective NP‐Score of pseudo‐NPs can be explained by the fact that their design is based on unprecedented combinations of NP‐derived fragments. Thus, the particular connection pathways between atoms in pseudo‐NPs are different to an extent from those in NPs. As a result, pseudo‐NPs can be regarded as novel molecular matter, extending beyond the mere sum of their constituent parts.

Additionally, the NP‐Score distribution of pseudo‐NPs has significant overlap with the NP‐Score distribution of DrugBank compounds. Further analysis of the molecular properties of pseudo‐NPs, in particular molecular weight and calculated lipophilicity, showed that most pseudo‐NPs (67 %) fall within the “rule‐of‐five” space^[49]^ (Figure 3, top right). This observation extends to additional metrics such as total polar surface area^[50]^ and fraction of sp^3^‐hybridised carbons^[26]^ (Table S1), suggesting that pseudo‐NPs may be inherently endowed with desirable physiochemical properties. Furthermore, the shape diversity of the pseudo‐NP collection was assessed by calculating the molecules’ three principal moments of inertia (PMI).^[51]^ This assessment enables the direct evaluation of a molecule's shape in three‐dimensional space. The results (Figure 3, bottom left) show that pseudo‐NPs have a high degree of three‐dimensional character, and their distribution in the triangular plot is not congested along the rod‐disc‐like axis as observed with compound collections deriving from combinatorial design approaches.^[52]^ The distribution of pseudo‐NPs in the PMI plot is also similar to a set of selected NPs and non‐naturally occurring bioactive compounds (Figure 3, bottom right, see SI, Scheme S7 for structures), further demonstrating the high degree of shape diversity among different pseudo‐NP collections.

## 5 Biological Evaluation of Pseudo‐NP Libraries

**Figure 4:**
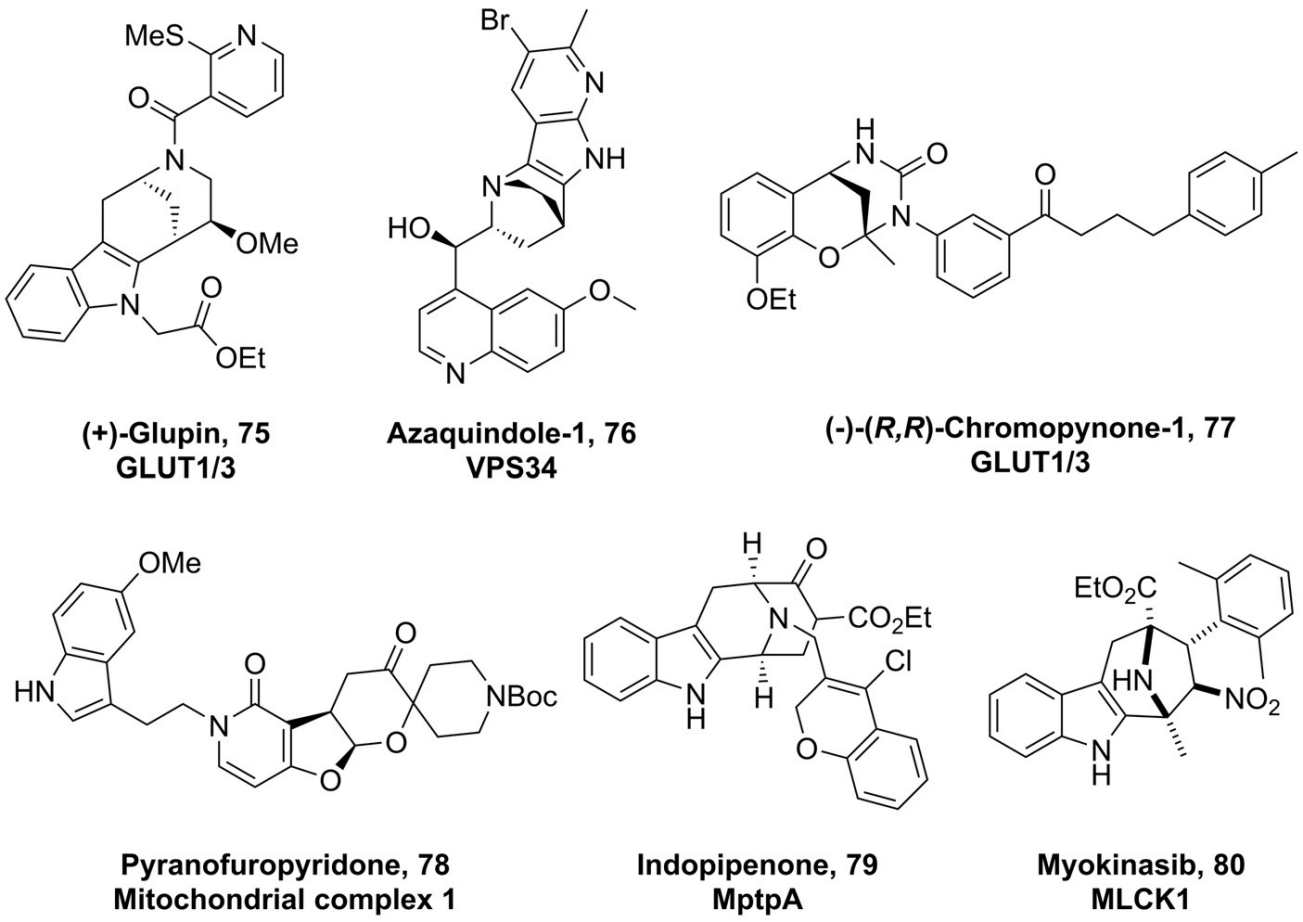
Structures of bioactive pseudo‐NPs and their molecular targets. Pseudo‐NPs display a diverse range of biological activities ranging from metabolic (GLUT inhibition) to anti‐microbial (MptpA) related targets. GLUT=glucose transporter; VPS34=vacuolar protein sorting 34; MLCK1=myosin light chain kinase 1.

To determine the utility of pseudo‐NPs systematically, broad biological screening is recommended. In this context, unbiased phenotypic screens offer an advantage over target‐based screens due to their greater coverage of biological space. This occurs because typically any given phenotype can be affected by the modulation of multiple macromolecular targets. All pseudo‐NPs we have produced have been screened in cell‐based assays monitoring glucose uptake, autophagy, Wnt and hedgehog signalling pathway activity, and induction of reactive oxygen species (ROS). Importantly, modulators of these therapeutically relevant phenotypes were prevalent amongst pseudo‐NPs. For example, chromopynone^[19]^ and indomorphan^[34]^ pseudo‐NPs such as **77** and **75** (Figure 4) were identified as highly potent inhibitors of glucose uptake by targeting the glucose transporters GLUT1 and GLUT3. Both are unprecedented chemotypes for GLUT inhibition. Furthermore, the 7‐azaindole‐fused quinine derivatives^[40]^ such as **76** (Figure 4) were highly potent inhibitors of starvation‐ and rapamycin‐induced autophagy. Importantly, the appropriate combination of NP fragments was essential for GLUT and autophagy inhibition, as none of the individual fragments possessed this activity. Finally indotropanes including Myokinasib (Figure 4, **80**), inhibited correct cytokinesis by acting as ATP‐competitive inhibitors of the myosin light chain kinase 1.^[53]^ Crucially, Myokinasib represents an unprecedented and unexpected bioactivity profile, as it is the first MLCK1 inhibitor reported, and a novel kinase inhibitory chemotype.

**Figure 5:**
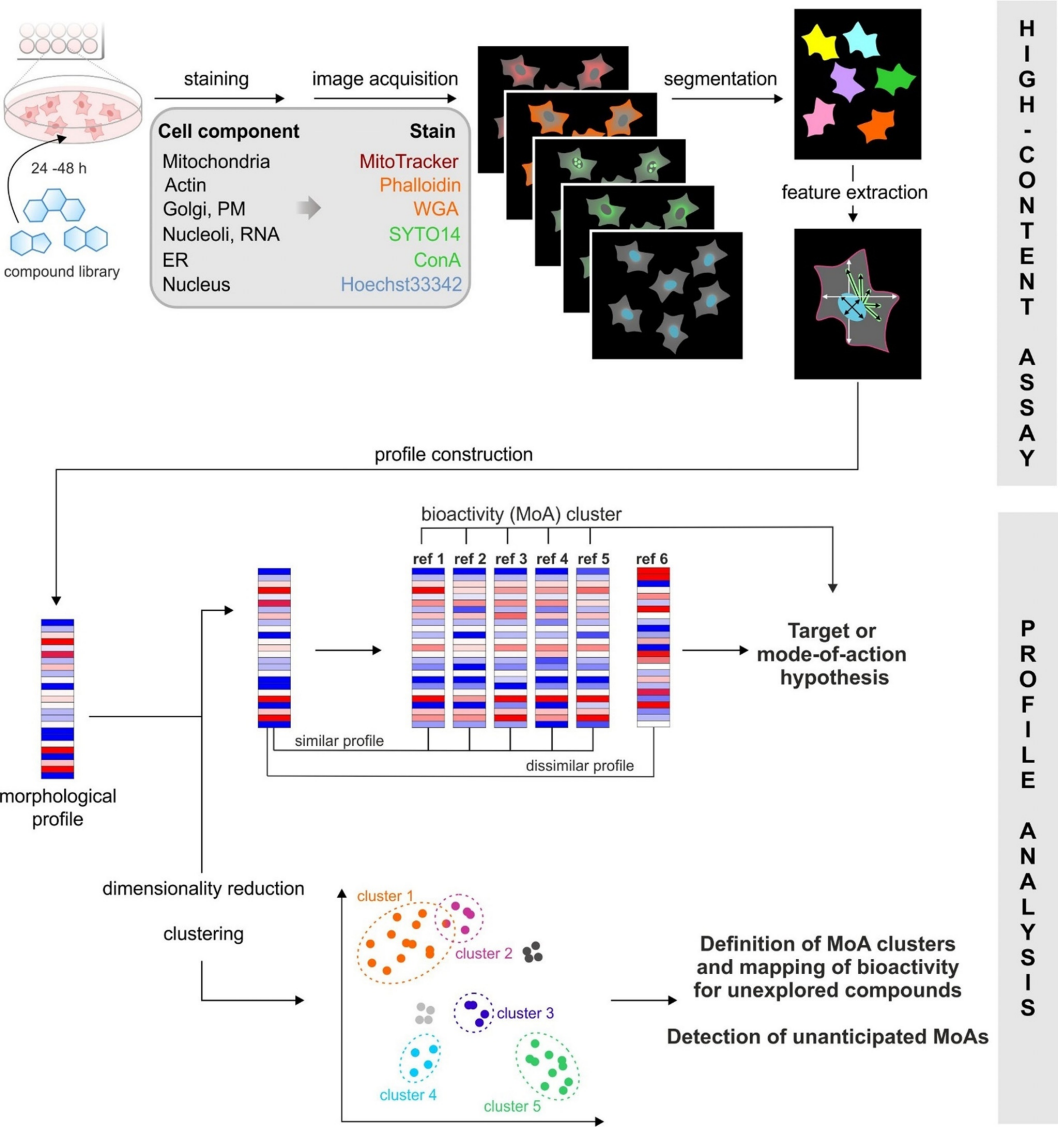
Morphological profiling using the cell painting assay. Cells are incubated with test compounds before being fixed and stained with dyes for different cellular components. Automated image acquisition and analysis allows morphological fingerprints to be generated for each small molecule. These can be compared to generate target or mode‐of‐action hypotheses, as well as clustering of bioactive molecules. Adapted from Ziegler et al.^[56]^.

In addition to screens monitoring specific cellular phenotypes, a high‐content multiparametric imaging approach termed the cell painting assay (CPA)^[54]^ can be used to determine bioactivity in a broad sense (Figure 5). By staining different organelles with fluorescent dyes and imaging in five fluorescent channels, a multitude of parameters related to cellular morphology can be probed simultaneously.[^55^, ^56^] These can be used to generate fingerprints specific to a given treatment condition; for example, incubation with a compound. Compounds that induce a significant change compared to controls, as assessed by a so‐called “induction value” or alternatively by the Mahalanobis distance, are classed as bioactive.

For each of the different connectivity/regioisomeric arrangements of the pyrroquinolines (Scheme 2 d), distinct phenotypic outcomes were observed in the CPA.^[38]^ This work showed that differential combination of a confined set of NP‐fragments can provide scaffolds that exhibit diverse biological activity patterns. Morphological screening of the pseudo‐NP library described in Scheme 3 in the CPA revealed that a large proportion of compounds were bioactive. Notably, and as expected for pseudo‐NPs, the griseofulvin‐ and Cinchona alkaloid‐derived compounds were shown to have significantly different bioactivity profiles compared to their parent natural products. Investigation of the scaffolds and their bioactivity profiles by principal component analysis revealed that, in general, alternative combinations of the different fragments generated disparate biological effects, as indicated by their different phenotypic profiles. The fragments from which these pseudo‐NPs were constructed, therefore, do not dominate bioactivity of the new combination, and can be considered favourable choices for the design and synthesis of further pseudo‐NP classes with novel fragment combinations. Notably, however, compounds derived from the sinomenine NP‐fragment generally displayed highly similar phenotypic profiles, suggesting a dominating biological effect by the imbedded morphine‐type scaffold, such that the sinomenine fragment may not be a favourable choice for additional fragment combinations aiming at different bioactivity. Indeed, these insights enabled prospective design of pseudo‐NP collections with alternative bioactivity profiles.

As well as identifying bioactivity in a general sense, the CPA has the additional advantage of potentially identifying molecular targets or predicting compound mode‐of‐action by comparing the fingerprint produced by a novel small molecule to those of an annotated reference set. Furthermore, non‐dominant fragments can be identified in this manner, which can provide guidance for the design of subsequent pseudo‐NP classes with potentially new biological activity (see Section 5). Crucially, and unlike chemoproteomic target identification strategies, the CPA can identify targets and modes‐of‐action that occur as a result of the modulation of a non‐protein target. For example, it is an excellent tool for identifying compounds that interfere with lysosomal activity,^[57]^ and metal ion chelators.^[58]^ In the context of pseudo‐NPs, the CPA was able to identify the target of pyrano‐furo‐pyridones as mitochondrial complex I due to the high biosimilarity of these compounds with the reported complex I inhibitor aumitin.^[59]^ The target of the azaindole‐fused quinine, Azaquindole‐1, was identified as the lipid kinase VPS34 due to the high biosimilarity of this compound to the selective VPS34 inhibitor SAR405.

## 6 Chemical Evolution of NP Structure

NPs are biologically relevant because they can interact with proteins which, for example, serve as receptors, or enzymes, and have co‐evolved together with specifically binding small molecules. New or altered NPs emerge by natural evolution. They are the product of coordinated enzymatic cascades which, in turn, result from regulated gene clusters. During organismal replication, alterations such as gene recombination, duplication, or mutation occur within these clusters which lead to modified enzymes and thereby to altered NPs. A typical scenario for the consequences of recombination and mutation is that, in a certain organism which executes the synthetic strategy for a new NP, binding between a NP and a specific protein is enabled or improved, and consequently exerts a positive reproductive effect for exactly this organism. Natural evolution can therefore be regarded as a gigantic “process of learning by matter” which is based on a simple algorithm as well as the requirement that every target molecule, cell, or organism can be described by the *information* which is passed on to descendants. The *genetic information* of every living organism or, their *genotype*, is “encoded” by the linear copolymers of DNA and RNA, and it is “expressed” in proteins which form the entirety of properties of this genotype, the *phenotype* and target of selection.

The evolutionary algorithm has long been exploited for the “directed evolution of biomolecules” such as RNA or proteins,^[60]^ and specifically the tailoring of naturally occuring enzymes for specific purposes.^[61]^ However, the principle of evolution can also accelerate the development of NPs and lead to pseudo‐NPs. When considering evolutionary optimisation of NPs, we need to rethink the terms genotype and phenotype: Each small molecular structure encodes *chemical information* that is the sum of informational contributions stored within the chemical microenvironment of every individual atom of a molecule.^[62]^ From this perspective, NP‐derived fragments consist of connected informational units similar to DNA or RNA and represent individual genotypes. Their chemical information is densely packed and encompasses high fractions of sp^3^‐hybridized atoms, high stereogenic content, high heteroatom content, and low aromaticity.[^62^, ^63^] Each genotype determines a three‐dimensional structure with specific physico‐chemical and biochemical characteristics, that is, the genotype is expressed as a phenotype. The phenotype, in turn, can form complementary interactions with other molecules and, consequently, serve as the target of selection and evolutionary optimisation. A similar genotype–phenotype dichotomy is known for RNA.

**Figure 6:**
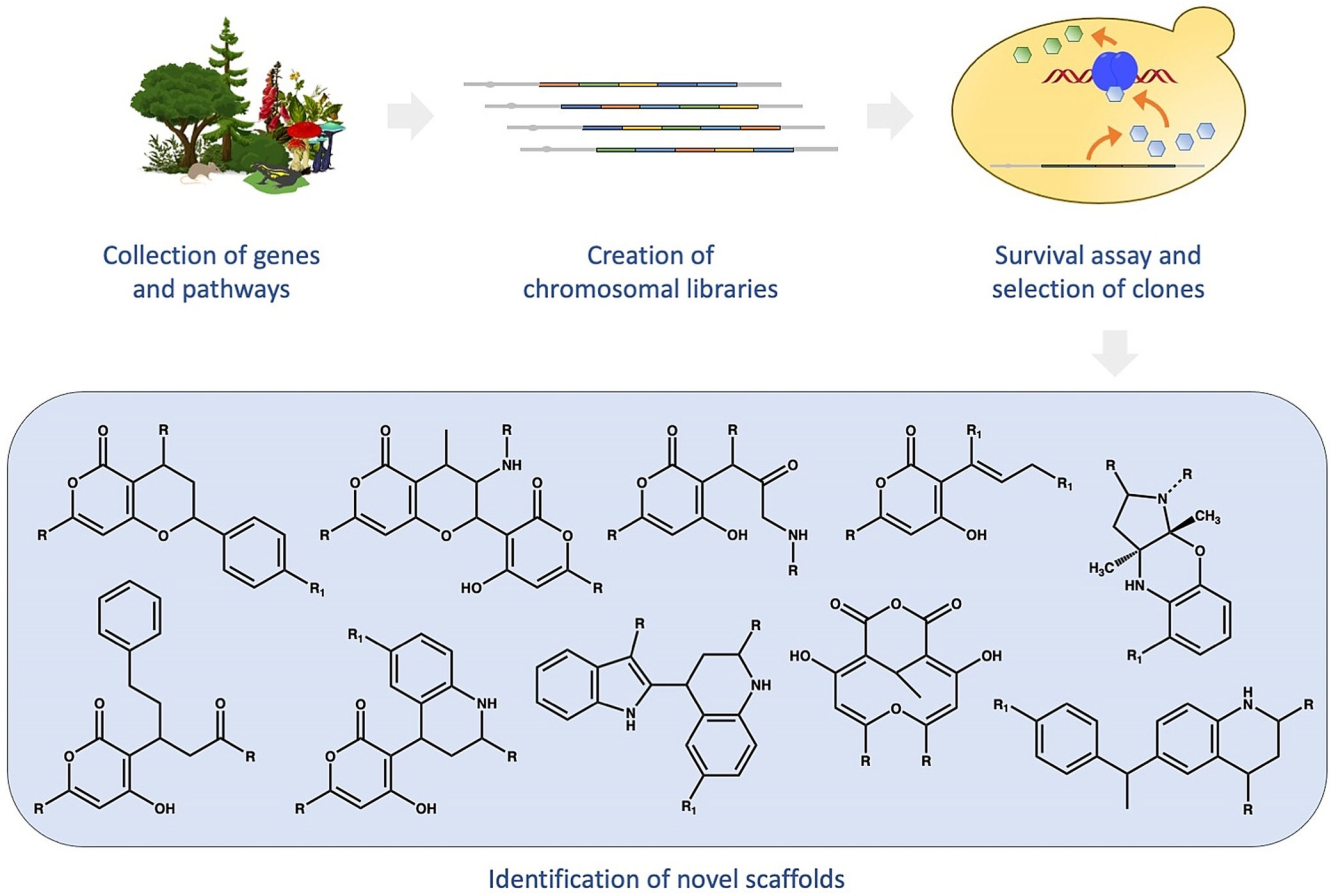
Scheme of the “synthetic biology combinatorial genetics” approach developed by Evolva.^[65]^ Briefly, the procedure starts with the collection and cloning of genetic material encoding biosynthetic pathways together with cDNA libraries from diverse natural sources. After recombination and expression in a microbial host, additional or altered enzymes will supplement or modify existing pathways, thereby enabling the synthesis of new or modified natural products. Their presence and possible action in vivo can be challenged in cellular assays in which surviving clones may reveal a “fitter” or, simply, altered behaviour. Clones are sorted according to selective criteria and submitted to a range of preparative and analytic procedures for obtaining and identifying small molecules.

The way in which previously unknown NPs that combine known fragments in a new or uncommon way can arise, was recently shown in a landmark study: For this, genetic material encoding enzymatic cascades from different sources and cDNA libraries of diverse organisms was recombined in a microbial host and submitted to selective constraints requiring the action of target proteins in a survival assay (Figure 6).[^64^, ^65^] From surviving cells, 74 novel chemical structures were isolated, more than 75 % of which had not been described so far. Their detailed inspection revealed that a fraction of these compounds emerged from hitherto unknown combinations of known NP‐derived substructures (i.e. combination of substructures for the first time), as well as new combinations of known natural product‐derived substructures (i.e. combinations of substructures in new connectivities).^[65]^ This finding suggests that novel combinations of NP fragments in nature can be induced, and that the current repertoire of known NPs, in principle, can be complemented by existing, but maybe currently not actively or differently used biosynthetic pathways. By analogy, NP fragments could be employed as “inheritable building blocks” in a new, evolutionary strategy towards bioactive compound discovery. In this strategy, the novel combination of NP‐fragments by biosynthetic steps as described above would be replaced by synthetic fragment combinations leading to pseudo‐natural products. These pseudo‐NPs currently have not been identified from natural sources, but the example described above suggests that, in principle, they may be amenable to biosynthesis. Also, it is possible that they may indeed exist in nature but have not been identified yet. In fact, after the pyrroquinoline scaffold **38** had been synthesised in the context of a pseudo‐NP program,^[38]^ the scaffold was reported to occur in nature in the NP Albogrisin.^[66]^

Evolutionary optimisation requires that genotypes are varied by *recombination* and/or *mutation*, and their phenotypes are sorted under specific constraints. The best‐performing phenotypes (“the fittest”) are selected because they confer an advantage, for example, improved molecular recognition or effector features. Since the fittest descendants have a genotype (=hereditary information) that was not present before, evolution can also be understood as a process during which new information is continually generated.

**Figure 7:**
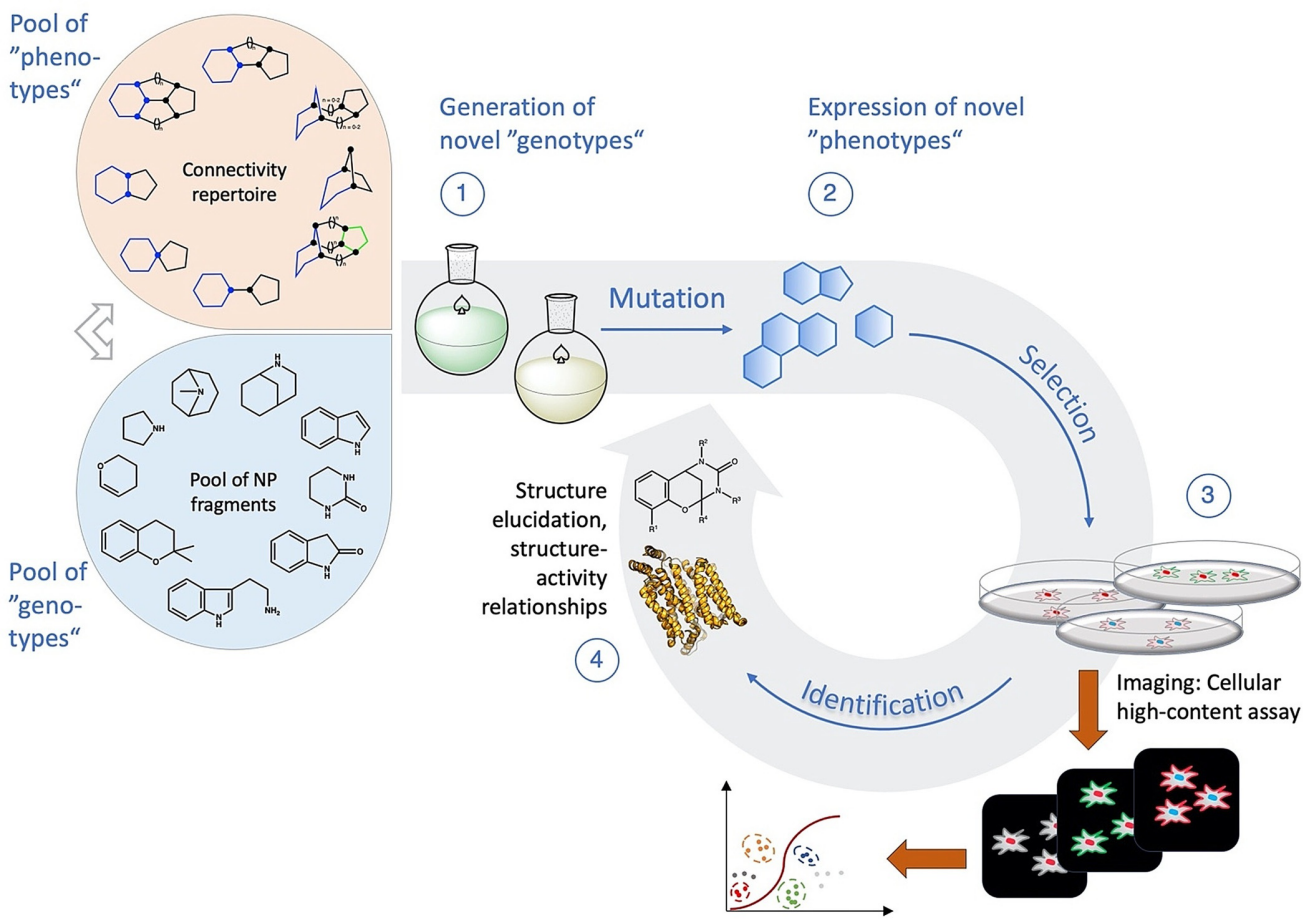
Focused chemical evolution. (1) The cyclic procedure starts with the generation of a “pool of genotypes” which is synthesised from NP fragments (=inheritable chemical information) using synthetic strategies that allow for the combinatorial application of a set of feasible connectivity patterns as well as further derivatisations. (2) Structural properties of the resulting compound library, such as content of sp^3^‐hybridised atoms, stereocentres, heteroatoms, and aromaticity, account for the expression of “phenotypes”—the potential to interact with specific structural motifs of proteins. (3) The compound library then is applied to a cellular screening platform (e.g., yeast cells) which is optically monitored for structural changes, e.g., by fluorescence imaging. Data are combined, analysed, and sorted according to selective constraints. (4) Molecules causing a desired change in the cellular system are isolated and identified (NMR, MS) and submitted to further structural characterisation, for example, by co‐crystallisation with putative target proteins (example used here: 4PYP^[67]^). The outcome of this round is the beginning of a new cycle which includes the “new chemical information” that was received.

As shown below (Figure 7), the design and synthesis of pseudo‐NPs starts from a pool of biologically relevant fragments, corresponding to a set of genotypes. Their variation (mutation) is achieved using synthetic strategies which consist of fragment assembly by recombination (Figure 2; connectivity patterns) and further derivatisation. The resulting compound library is a pool of new genotypes which express new phenotypes, represented by their (three‐dimensional) structures which confer the ability to recognise and bind target proteins. When exposed to a biological system such as a cell culture, some phenotypes eventually reveal new biological activity, for example, by perturbing vital processes or modifying biomarkers. Selection takes place when these new pseudo‐NPs are identified, isolated and characterised, the latter serving for recognition and understanding of the newly generated chemical information. Following nature's example, the complete process may be repeated in a circular manner, where the output of a previous cycle serves as the input for a subsequent cycle (Figure 7). In this sense, the process of: i) designing, ii) preparing, and iii) biologically characterising pseudo‐NPs, resulting in new information (both chemical and biological), which subsequently initiates iterative process cycles, can be regarded as a chemical evolution of natural product structure.

## 7 Discussion

The discovery of bioactive small molecules that can modulate biological processes in a selective and time‐resolved manner, as well as their efficient preparation, can have great impact on the understanding of biology and disease, and thus provide new therapeutic opportunities.^[2]^ Historically, NPs have provided ample inspiration for the design of new biologically relevant compounds.^[46]^

Several approaches have been developed exploiting NP structures in distinct ways. More specifically, biology‐oriented synthesis (BIOS), exploits substructures of NP scaffolds to prepare compound collections that inherit the biological relevance of NPs.[^12^, ^13^] Alternatively, “complexity‐to‐diversity” (CtD), exploits low‐to‐medium molecular weight NPs amenable to chemoselective processes as starting materials for the preparation of diverse and biologically relevant molecular scaffolds.[^68^, ^69^, ^70^] However, both of these approaches may be limited in the extent of the exploration of chemical space that they can offer. Additionally, the observed biological activity of compound classes directly delineated from existing NPs may not differ significantly from that of the guiding NP structure, and thus limits the range of biological space that can be interrogated by them.

In an effort to mitigate these limitations a new approach has been developed, involving the fusion of NP‐derived fragments in unprecedented combinations, affording novel biologically relevant molecular structures termed pseudo‐natural products (pseudo‐NPs). This approach builds on the biological relevance of NPs and the efficient exploration of chemical space offered by combinations of fragment‐sized compounds. In this context the original “rule‐of‐three” definition of fragments was relaxed since it may not be entirely valid for natural products, and as a filter for fragment likeness AlogP≤3.5, MW 120–350 Da, ≤3 hydrogen bond donors, ≤6 hydrogen bond acceptors, and ≤6 rotatable bonds were chosen. Earlier proof‐of‐concept studies have demonstrated the viability of the approach and produced a set of guidelines for the design of new structures.[^19^, ^20^] For example, two NP‐derived fragments can be connected through different connectivity patterns (Figure 2, Panel A), to produce different pseudo‐NP classes, each of which has been shown to occupy different regions of chemical space (Figure 2, Panel C, Design Principle 1).^[20]^ Additional pseudo‐NP classes can be designed by maintaining the same connectivity pattern through different connection points on one or both fragments (Figure 2, Panel C, Design Principle 2). These guidelines demonstrate the great potential for the preparation of novel molecular scaffolds, which may be inherently biological relevant, limited only by human imagination and the availability of synthetic methods.

The preparation of pseudo‐NP classes such as pyrroquinolines (Scheme 2 i) demonstrated in practice that the combination of a set of common NP‐derived fragments in different connectivity patterns (exploiting Design Principle 1) can yield chemically and biologically diverse libraries. Additionally, this work also demonstrated that these regioisomeric pseudo‐NP classes display distinct biological effects. Furthermore, combinations of different fragments in complementary arrangements (exploiting Design Principle 2) can produce chemically and biologically diverse compound libraries (Scheme 3).[^40^, ^41^] As such, pseudo‐NPs constitute novel chemical matter and possess more than just the additive properties of their individual constituting fragments. This observation is also strongly supported by the biological activity observed for different pseudo‐NP classes. For example, the activity of pseudo‐NPs, such as the chromopynones,^[19]^ pyrano‐furo‐pyridones,^[33]^ indomorphans,^[34]^ and azaquindoles^[40]^ was not shared by either of the individual NP‐derived fragments.

This inherited biological relevance from NPs, to NP‐derived fragments, to pseudo‐NPs, suggests a continuation of biologically relevant chemical space. In nature, entry points to this space created through evolution convey an advantage to the host organism and usually are not re‐optimised. Instead, they are used in different arrangements. Thus, such biologically relevant portions of chemical space may only be accessible by exploiting features encoded and subsequently passed‐on through the incorporation of structural motifs generated by secondary metabolite biosynthesising proteins, which themselves share evolutionary conserved structural features.^[13]^ This hypothesis is supported by the fact that NPs interact with multiple proteins during their biosynthesis and must be thus endowed with several biologically relevant structural motifs. Additionally, recent reports make a case for a more prevalent role of active transport in the cellular uptake of small molecules.^[71]^ Work by Kell and co‐workers advocates that small molecule cellular transport occurs through specific interactions with membrane proteins.[^72^, ^73^, ^74^] Such a mechanism would require the existence of distinct biologically relevant structural motifs to be present in bioactive molecules, independent of their ultimate molecular properties such as molecular weight or lipophilicity.

The finding that novel combinations of NP fragments can be induced by recombination of existing biosynthesis pathways suggests that the current repertoire of known NPs, in principle, can be extended through synthetic biology techniques. This may be achieved, for instance, by changes to natural biosynthesis to make use of different existing biosynthesis pathways, including “silent” pathways that are not usually active. By analogy, synthetic chemical recombination of NP fragments as “inheritable building blocks” defines an evolutionary strategy for the discovery of novel natural product‐inspired compound classes a priori endowed with biological relevance. In this strategy the novel combination of NP‐fragments by biosynthetic steps is replaced by unprecedented synthetic NP‐fragment combinations. Thus, the iterative design, synthesis, and biological investigation of pseudo‐NPs can be regarded as a chemical evolution of natural product structure, and as a human and chemically‐driven branch of the evolution of NPs (Figure 7).

**Figure 8:**
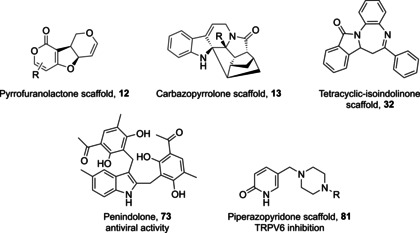
Structures of independently reported pseudo‐natural products. Specific biological activity has already been attributed to penindilones such as **73** and piperazopyridinones (scaffold **81**). Other scaffolds such as **12**, **13**, or **32** may be biologically active, however, no specific activity has been attributed to them to date.

In order to investigate the generality of these principles, we also conducted a literature search for further examples of molecules that may satisfy their classification as pseudo‐NPs. This search revealed pseudo‐NPs prepared independently by different research groups. Notable examples include the pyrrofuranolactones **12**,^[31]^ carbazopyrrolones **13**,^[32]^ as well as the more recent penindolones **71**–**74**,^[42]^ piperazopyridones **81**,^[75]^ and tetracyclic‐fused isoindolinones **32** (Figure 8).^[35]^ Some of these molecules have displayed biological activity. For example, penindolones were found to inhibit membrane fusion of the Influenza A virus,^[42]^ and derivatives of piperazopyridones were identified as TRPV6 channel inhibitors.^[75]^ Other applications include the preparation of pseudo‐NP dyes from betelamic acid.^[76]^ These examples indicate that the concept might have been intuitively employed previously, without the intellectual framework and guidance of the design principles delineated here.

## 8 Outlook

Pseudo‐NPs stem from combinations of NP‐derived fragments leading to molecular scaffolds that may not be accessible by natural biosynthesis. These structures combine the ability of fragments to rapidly explore chemical space with the biological relevance of NPs, resulting in a molecular discovery strategy that can uncover unexplored regions of biologically relevant chemical space potentially harbouring compounds with unprecedented biological activity. Through the case studies highlighted above it is evident that pseudo‐NPs represent a validated general design approach for molecular discovery, and can generate numerous opportunities for chemical biology and medicinal chemistry research.

The design of pseudo‐NPs takes into account general molecular properties, such as Fsp^3^ (fraction of sp^3^‐hybridised carbon atoms) and heteroatom content, as well as the more specific principle of joining individual fragments based on connectivity patterns observed in known NPs. As such, this chemocentric approach may be regarded as a chemically driven branch of the evolution of NPs. These design principles have been exploited by the scientific community as shown by selected examples above, yet not in a methodical manner. Potentially numerous compound classes which fall under the definition of pseudo‐NPs may already be known and can be analysed and assessed for biological activity. This evaluation would benefit from the application of multi‐parametric screening methods and may highlight applications of pseudo‐NPs in challenging areas such as developing antibiotics to combat multidrug resistant bacteria.^[60]^

Finally, pseudo‐NP design and preparation are underpinned by organic synthesis. The tools and methodologies developed for the preparation of NPs are certainly applicable in pseudo‐NPs as well. However, new synthetic methodologies,^[77]^ for example, photochemical C−H functionalisations,[^78^, ^79^] and creative scaffold‐forming multicomponent reactions[^80^, ^81^] will continue to have great impact on our ability to prepare and study further examples of these exciting and potentially highly beneficial compound classes.

## Conflict of interest

The authors declare no conflict of interest. G.K. is now an employee of AstraZeneca, U.K.

## Biographical Information

*George Karageorgis was born in Nicosia, Cyprus, and graduated from the Aristotle University of Thessaloniki, Greece, with a B.Sc. Chemistry degree in 2010. He obtained a M.Sc. in Chemical Biology from the University of Leeds in 2011, and then joined Prof. A. Nelson's group in Leeds as a PhD student where he worked on the development of activity‐directed synthesis. He earned an Alexander von Humboldt Fellowship and joined Prof. H. Waldmann's group in 2015 at the Max Planck Institute in Dortmund, working on the design and syntheses of biologically relevant small molecules with novel molecular scaffolds*.

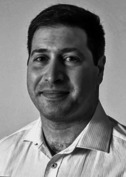

## Biographical Information

*Dan Foley carried out his PhD with Profs. Steve Marsden and Adam Nelson at Univ. Leeds (2015), then completed an EPRSC Doctoral Prize Fellowship (2015–2017). He carried out further postdoctoral studies (2017–2018) with Prof. Herbert Waldmann at the MPI of Molecular Physiology, where he held a Marie Skłodowska‐Curie Fellowship. Dan recently joined the faculty at the University of Canterbury, New Zealand, where his research focuses on the development of new synthetic methods of value to molecular discovery and medicinal chemistry*.

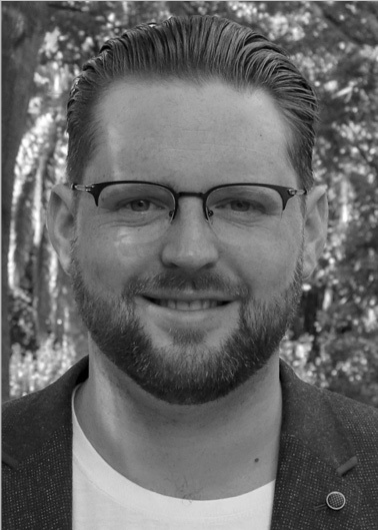

## Biographical Information

*Luca Laraia studied chemistry at Imperial College London, before moving to the University of Cambridge to carry out his Cancer Research UK‐funded PhD in chemical biology with Prof. David R. Spring and Prof. Ashok R. Venkitaraman. After graduating in 2014 he moved to the Max Planck Institute of Molecular Physiology (Dortmund, Germany), first as an Alexander von Humboldt postdoctoral fellow and then as project leader in the chemical biology department with Prof. Herbert Waldmann. He moved to DTU in November 2017 to take up an Assistant Professorship in chemical biology*.

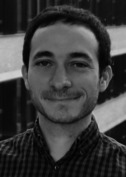

## Biographical Information

*Susanne Brakmann studied Chemistry at the TU Braunschweig (diploma, 1988) and received her Ph.D. from the University of Karlsruhe (with Reinhold Tacke, 1991). In 1992, she moved to the MPI for Biophysical Chemistry in Göttingen as a postdoctoral researcher with Manfred Eigen. During the period of 1999 to 2000, she applied her knowledge in an employment at Evotec Biosystems AG and in 2001, moved to the University of Leipzig as a junior research group leader. She received her habilitation from the TU Braunschweig in 2004 before she joined the TU Dortmund's Faculty of Chemistry and Chemical Biology*.

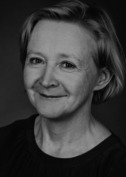

## Biographical Information

*Herbert Waldmann obtained his PhD in organic chemistry in 1985 under the supervision of Horst Kunz. After a postdoctoral period with George Whitesides at Harvard University, he returned to the University of Mainz and completed his habilitation in 1991. He was appointed as Director at MPI Dortmund and professor of Biochemistry at TU Dortmund University in 1999. His research focuses on new principles for the design and syntheses of natural‐product‐inspired compound classes and their biological evaluation*.

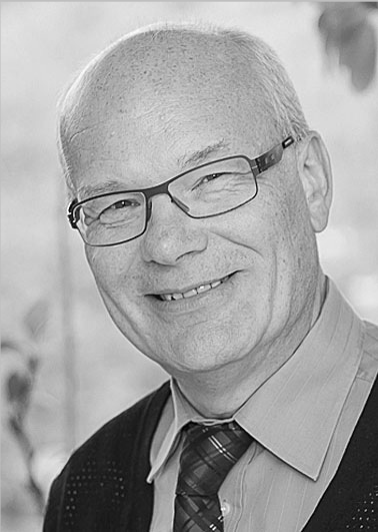

## Supporting information

As a service to our authors and readers, this journal provides supporting information supplied by the authors. Such materials are peer reviewed and may be re‐organized for online delivery, but are not copy‐edited or typeset. Technical support issues arising from supporting information (other than missing files) should be addressed to the authors.

Supplementary

## References

[1] M. S. Kinch, Drug Discovery Today 2014, 19, 1831–1835. 25172803 10.1016/j.drudis.2014.08.007

[2] A. M. Edwards, R. Isserlin, G. D. Bader, S. V. Frye, T. M. Willson, F. H. Yu, Nature 2011, 470, 163. 21307913 10.1038/470163a

[3] C. M. Dobson, Nature 2004, 432, 824–828. 15602547 10.1038/nature03192

[4] S. R. Langdon, N. Brown, J. Blagg, J. Chem. Inf. Model. 2011, 51, 2174–2185. 21877753 10.1021/ci2001428 PMC3180201

[5] S. L. Schreiber, Nature 2009, 457, 153–154. 19129834 10.1038/457153a

[6] H. S. G. Beckmann, F. Nie, C. E. Hagerman, H. Johansson, Y. S. Tan, D. Wilcke, D. R. Spring, Nat. Chem. 2013, 5, 861–867. 24056343 10.1038/nchem.1729

[7] M. D. Burke, S. L. Schreiber, Angew. Chem. Int. Ed. 2004, 43, 46–58; 10.1002/anie.200300626 14694470

[8] W. R. J. D. Galloway, A. Isidro-Llobet, D. R. Spring, Nat. Commun. 2010, 1, 80. 20865796 10.1038/ncomms1081

[9] E. Comer, J. A. Beaudoin, N. Kato, M. E. Fitzgerald, R. W. Heidebrecht, M. duPont Lee, D. Masi, M. Mercier, C. Mulrooney, G. Muncipinto, et al., J. Med. Chem. 2014, 57, 8496–8502. 25211597 10.1021/jm500994n PMC4207553

[10] F. G. Kuruvilla, A. F. Shamji, S. M. Sternson, P. J. Hergenrother, S. L. Schreiber, Nature 2002, 416, 653–657. 11948353 10.1038/416653a

[11] D. J. Newman, G. M. Cragg, J. Nat. Prod. 2016, 79, 629–661. 26852623 10.1021/acs.jnatprod.5b01055

[12] M. A. Koch, A. Schuffenhauer, M. Scheck, S. Wetzel, M. Casaulta, A. Odermatt, P. Ertl, H. Waldmann, Proc. Natl. Acad. Sci. USA 2005, 102, 17272–17277. 16301544 10.1073/pnas.0503647102 PMC1297657

[13] S. Wetzel, R. S. Bon, K. Kumar, H. Waldmann, Angew. Chem. Int. Ed. 2011, 50, 10800–10826; 10.1002/anie.201007004 22038946

[14] E. A. Crane, K. Gademann, Angew. Chem. Int. Ed. 2016, 55, 3882–3902; 10.1002/anie.201505863 PMC4797711 26833854

[15] D. A. Erlanson, S. W. Fesik, R. E. Hubbard, W. Jahnke, H. Jhoti, Nat. Rev. Drug Discovery 2016, 15, 605–619. 27417849 10.1038/nrd.2016.109

[16] H. Vu, L. Pedro, T. Mak, B. McCormick, J. Rowley, M. Liu, A. Di Capua, B. Williams-Noonan, N. B. Pham, R. Pouwer, et al., ACS Infect. Dis. 2018, 4, 431–444. 29436819 10.1021/acsinfecdis.7b00197 PMC5902791

[17] H. Prescher, G. Koch, T. Schuhmann, P. Ertl, A. Bussenault, M. Glick, I. Dix, F. Petersen, D. E. Lizos, Bioorg. Med. Chem. 2017, 25, 921–925. 28011199 10.1016/j.bmc.2016.12.005

[18] B. Over, S. Wetzel, C. Grütter, Y. Nakai, S. Renner, D. Rauh, H. Waldmann, Nat. Chem. 2013, 5, 21–28. 23247173 10.1038/nchem.1506

[19] G. Karageorgis, E. S. Reckzeh, J. Ceballos, M. Schwalfenberg, S. Sievers, C. Ostermann, A. Pahl, S. Ziegler, H. Waldmann, Nat. Chem. 2018, 10, 1103–1111. 30202104 10.1038/s41557-018-0132-6

[20] G. Karageorgis, D. J. Foley, L. Laraia, H. Waldmann, Nat. Chem. 2020, 12, 227–235. 32015480 10.1038/s41557-019-0411-x

[21] T. Ozaki, K. Yamashita, Y. Goto, M. Shimomura, S. Hayashi, S. Asamizu, Y. Sugai, H. Ikeda, H. Suga, H. Onaka, Nat. Commun. 2017, 8, 14207. 28165449 10.1038/ncomms14207 PMC5303826

[22] Y. Goto, Y. Ito, Y. Kato, S. Tsunoda, H. Suga, Chem. Biol. 2014, 21, 766–774. 24856821 10.1016/j.chembiol.2014.04.008

[23] H. Kikuchi, K. Ichinohe, S. Kida, S. Murase, O. Yamada, Y. Oshima, Org. Lett. 2016, 18, 5948–5951. 27934494 10.1021/acs.orglett.6b03057

[24] T. Asai, K. Tsukada, S. Ise, N. Shirata, M. Hashimoto, I. Fujii, K. Gomi, K. Nakagawara, E. N. Kodama, Y. Oshima, Nat. Chem. 2015, 7, 737–743. 26291946 10.1038/nchem.2308

[25] M. Feher, J. M. Schmidt, J. Chem. Inf. Comput. Sci. 2003, 43, 218–227. 12546556 10.1021/ci0200467

[26] F. Lovering, J. Bikker, C. Humblet, J. Med. Chem. 2009, 52, 6752–6756. 19827778 10.1021/jm901241e

[27] F. Lovering, MedChemComm 2013, 4, 515–519.

[28] S. P. Kureel, R. S. Kapil, S. P. Popli, Tetrahedron Lett. 1969, 10, 3857–3862. 10.1016/s0040-4039(01)88531-2 5348311

[29] G. Palmisano, R. Annunziata, G. Papeo, M. Sisti, Tetrahedron: Asymmetry 1996, 7, 1–4.

[30] L. Ding, A. Maier, H.-H. Fiebig, W.-H. Lin, C. Hertweck, Org. Biomol. Chem. 2011, 9, 4029–4031. 21528153 10.1039/c1ob05283g

[31] M. J. Bartlett, C. A. Turner, J. E. Harvey, Org. Lett. 2013, 15, 2430–2433. 23621816 10.1021/ol400902d

[32] B. de Carné-Carnavalet, J.-P. Krieger, B. Folléas, J.-L. Brayer, J.-P. Demoute, C. Meyer, J. Cossy, Eur. J. Org. Chem. 2015, 2015, 1273–1282.

[33] A. Christoforow, J. Wilke, A. Binici, A. Pahl, C. Ostermann, S. Sievers, H. Waldmann, Angew. Chem. Int. Ed. 2019, 58, 14715–14723; 10.1002/anie.201907853 PMC7687248 31339620

[34] J. Ceballos, M. Schwalfenberg, G. Karageorgis, E. S. Reckzeh, S. Sievers, C. Ostermann, A. Pahl, M. Sellstedt, J. Nowacki, M. A. Carnero Corrales, et al., Angew. Chem. Int. Ed. 2019, 58, 17016–17025; 10.1002/anie.201909518 PMC6900016 31469221

[35] S. Yuan, Y.-L. Yue, D.-Q. Zhang, J.-Y. Zhang, B. Yu, H.-M. Liu, Chem. Commun. 2020, 56, 11461–11464. 10.1039/d0cc04875e 32853306

[36] Y.-C. Lee, S. Patil, C. Golz, C. Strohmann, S. Ziegler, K. Kumar, H. Waldmann, Nat. Commun. 2017, 8, 14043. 28195128 10.1038/ncomms14043 PMC5316858

[37] A. Nören-Müller, W. Wilk, K. Saxena, H. Schwalbe, M. Kaiser, H. Waldmann, Angew. Chem. Int. Ed. 2008, 47, 5973–5977; 10.1002/anie.200801566 18604796

[38] J. Liu, G. S. Cremosnik, F. Otte, A. Pahl, S. Sievers, C. Strohmann, H. Waldmann, Angew. Chem. Int. Ed. 2021, 60, 4648–4656; 10.1002/anie.202013731 PMC7986669 33200868

[39] A. H. Lipkus, Q. Yuan, K. A. Lucas, S. A. Funk, W. F. Bartelt, R. J. Schenck, A. J. Trippe, J. Org. Chem. 2008, 73, 4443–4451. 18505297 10.1021/jo8001276

[40] D. J. Foley, S. Zinken, D. Corkery, L. Laraia, A. Pahl, Y.-W. Wu, H. Waldmann, Angew. Chem. Int. Ed. 2020, 59, 12470–12476; 10.1002/anie.202000364 PMC7383971 32108411

[41] M. Grigalunas, A. Burhop, S. Zinken, A. Pahl, S. Sievers, D. J. Foley, A. P. Antonchick, H. Waldmann, *Nat. Commun*., 10.1038/s41467-021-22174-4. PMC7994817 33767198

[42] G. Wu, G. Yu, Y. Yu, S. Yang, Z. Duan, W. Wang, Y. Liu, R. Yu, J. Li, T. Zhu, et al., J. Med. Chem. 2020, 63, 6924–6940. 32520560 10.1021/acs.jmedchem.0c00312

[43] P. Ertl, S. Roggo, A. Schuffenhauer, J. Chem. Inf. Model. 2008, 48, 68–74. 18034468 10.1021/ci700286x

[44] D. S. Wishart, Y. D. Feunang, A. C. Guo, E. J. Lo, A. Marcu, J. R. Grant, T. Sajed, D. Johnson, C. Li, Z. Sayeeda, et al., Nucleic Acids Res. 2018, 46, D1074–D1082. 29126136 10.1093/nar/gkx1037 PMC5753335

[45] M. Davies, M. Nowotka, G. Papadatos, N. Dedman, A. Gaulton, F. Atkinson, L. Bellis, J. P. Overington, Nucleic Acids Res. 2015, 43, W612–W620. 25883136 10.1093/nar/gkv352 PMC4489243

[46] E. Patridge, P. Gareiss, M. S. Kinch, D. Hoyer, Drug Discovery Today 2016, 21, 204–207. 25617672 10.1016/j.drudis.2015.01.009

[47] B. L. DeCorte, J. Med. Chem. 2016, 59, 9295–9304. 27331414 10.1021/acs.jmedchem.6b00473

[48] T. Rodrigues, D. Reker, P. Schneider, G. Schneider, Nat. Chem. 2016, 8, 531. 27219696 10.1038/nchem.2479

[49] C. A. Lipinski, F. Lombardo, B. W. Dominy, P. J. Feeney, Adv. Drug Delivery Rev. 1997, 23, 3–25. 10.1016/s0169-409x(00)00129-0 11259830

[50] D. F. Veber, S. R. Johnson, H.-Y. Cheng, B. R. Smith, K. W. Ward, K. D. Kopple, J. Med. Chem. 2002, 45, 2615–2623. 12036371 10.1021/jm020017n

[51] W. H. B. Sauer, M. K. Schwarz, J. Chem. Inf. Comput. Sci. 2003, 43, 987–1003. 12767158 10.1021/ci025599w

[52] T. James, P. MacLellan, G. M. Burslem, I. Simpson, J. A. Grant, S. Warriner, V. Sridharan, A. Nelson, Org. Biomol. Chem. 2014, 12, 2584–2591. 24614952 10.1039/c3ob42512f

[53] T. Schneidewind, S. Kapoor, G. Garivet, G. Karageorgis, R. Narayan, G. Vendrell-Navarro, A. P. Antonchick, S. Ziegler, H. Waldmann, Cell Chem. Biol. 2019, 26, 512–523. 30686759 10.1016/j.chembiol.2018.11.014

[54] M.-A. Bray, S. Singh, H. Han, C. T. Davis, B. Borgeson, C. Hartland, M. Kost-Alimova, S. M. Gustafsdottir, C. C. Gibson, A. E. Carpenter, Nat. Protoc. 2016, 11, 1757–1754. 27560178 10.1038/nprot.2016.105 PMC5223290

[55] A. Pahl, S. Sievers, The Cell Painting Assay as a Screening Tool for the Discovery of Bioactivities in New Chemical Matter in Systems Chemical Biology (Eds.: S. Ziegler, H. Waldmann), Springer, New York, 2019, pp. 115–126. 10.1007/978-1-4939-8891-4_6 30519943

[56] S. Ziegler, S. Sievers, H. Waldmann, *Cell Chem. Biol*., 10.1038/s41467-021-22174-4. 33740434

[57] L. Laraia, G. Garivet, D. J. Foley, N. Kaiser, S. Müller, S. Zinken, T. Pinkert, J. Wilke, D. Corkery, A. Pahl, et al., Angew. Chem. Int. Ed. 2020, 59, 5721–5729; 10.1002/anie.201913712 PMC7154763 31769920

[58] T. Schneidewind, A. Brause, A. Pahl, A. Burhop, T. Mejuch, S. Sievers, H. Waldmann, S. Ziegler, ChemBioChem 2020, 21, 3197. 32618075 10.1002/cbic.202000381 PMC7754162

[59] L. Robke, Y. Futamura, G. Konstantinidis, J. Wilke, H. Aono, Z. Mahmoud, N. Watanabe, Y.-W. Wu, H. Osada, L. Laraia, et al., Chem. Sci. 2018, 9, 3014–3022. 29732085 10.1039/c7sc05040b PMC5916016

[60] K. Lewis, Cell 2020, 181, 29–45. 32197064 10.1016/j.cell.2020.02.056

[61] F. H. Arnold, Angew. Chem. Int. Ed. 2019, 58, 14420–14426; 10.1002/anie.201907729 31433107

[62] T. Böttcher, J. Chem. Inf. Model. 2016, 56, 462–470. 26857537 10.1021/acs.jcim.5b00723

[63] R. M. Demoret, M. A. Baker, M. Ohtawa, S. Chen, C. C. Lam, S. Khom, M. Roberto, S. Forli, K. N. Houk, R. A. Shenvi, J. Am. Chem. Soc. 2020, 142, 18599–18618. 32991152 10.1021/jacs.0c08231 PMC7727090

[64] M. Naesby, S. V. S. Nielsen, C. A. F. Nielsen, T. Green, T. Ø. Tange, E. Simón, P. Knechtle, A. Hansson, M. S. Schwab, O. Titiz, et al., Microb. Cell Fact. 2009, 8, 45–56. 19678954 10.1186/1475-2859-8-45 PMC2732597

[65] J. Klein, J. R. Heal, W. D. O. Hamilton, T. Boussemghoune, T. Ø. Tange, F. Delegrange, G. Jaeschke, A. Hatsch, J. Heim, ACS Synth. Biol. 2014, 3, 314–323. 24742115 10.1021/sb400177x PMC4046787

[66] G. S. Cremosnik, J. Liu, H. Waldmann, Nat. Prod. Rep. 2020, 37, 1497–1510. 33020792 10.1039/d0np00015a

[67] D. Deng, C. Xu, P. Sun, J. Wu, C. Yan, M. Hu, N. Yan, Nature 2014, 510, 121–125. 24847886 10.1038/nature13306

[68] R. W. Huigens III , K. C. Morrison, R. W. Hicklin, T. A. Flood Jr , M. F. Richter, P. J. Hergenrother, Nat. Chem. 2013, 5, 195–202. 23422561 10.1038/nchem.1549 PMC3965367

[69] V. M. Norwood IV , R. W. Huigens III , ChemBioChem 2019, 20, 2273–2297. 30609199 10.1002/cbic.201800768

[70] S. E. Motika, P. J. Hergenrother, Nat. Prod. Rep. 2020, 37, 1395–1403. 33034322 10.1039/d0np00059k PMC7720426

[71] E. Girardi, A. César-Razquin, S. Lindinger, K. Papakostas, J. Konecka, J. Hemmerich, S. Kickinger, F. Kartnig, B. Gürtl, K. Klavins, et al., Nat. Chem. Biol. 2020, 16, 469–478. 32152546 10.1038/s41589-020-0483-3 PMC7610918

[72] D. B. Kell, P. D. Dobson, S. G. Oliver, Drug Discovery Today 2011, 16, 704–714. 21624498 10.1016/j.drudis.2011.05.010

[73] S. O'Hagan, D. B. Kell, ADMET DMPK 2017, 5, 85–125.

[74] D. B. Kell, S. G. Oliver, Front. Pharmacol. 2014, 5, 1–32. 25400580 10.3389/fphar.2014.00231 PMC4215795

[75] M. R. Cunha, R. Bhardwaj, A. L. Carrel, S. Lindinger, C. Romanin, R. Parise-Filho, M. A. Hediger, J.-L. Reymond, RSC Med. Chem. 2020, 11, 1032–1040. 33479695 10.1039/d0md00145g PMC7513592

[76] R. M. Pioli, R. R. Mattioli, L. C. Esteves, S. Dochev, E. L. Bastos, Dyes Pigm. 2020, 183, 108609.

[77] D. C. Blakemore, L. Castro, I. Churcher, D. C. Rees, A. W. Thomas, D. M. Wilson, A. Wood, Nat. Chem. 2018, 10, 383–394. 29568051 10.1038/s41557-018-0021-z

[78] H. Yi, G. Zhang, H. Wang, Z. Huang, J. Wang, A. K. Singh, A. Lei, Chem. Rev. 2017, 117, 9016–9085. 28639787 10.1021/acs.chemrev.6b00620

[79] X. Lang, X. Chen, J. Zhao, Chem. Soc. Rev. 2014, 43, 473–486. 24162830 10.1039/c3cs60188a

[80] O. Ghashghaei, M. Pedrola, F. Seghetti, V. V. Martin, R. Zavarce, M. Babiak, J. Novacek, F. Hartung, K. M. Rolfes, T. Haarmann-Stemmann, et al., Angew. Chem. Int. Ed. 2021, 60, 2603–2608. 10.1002/anie.202011253 33048416

[81] S. Zhi, X. Ma, W. Zhang, Org. Biomol. Chem. 2019, 17, 7632–7650. 31339143 10.1039/c9ob00772e

