# Supplementary material for: Pseudo Natural Products—Chemical Evolution of Natural Product Structure
Source: Angew Chem Int Ed Engl. 2021 Mar 23;60(29):15705–23. doi: 10.1002/anie.202016575 (PMC8360037; doi:10.1002/anie.202016575)
Supplement: Supplementary file 1 — Supplementary [file ANIE-60-15705-s001.pdf]

Angewandte Chemie
Eine Zeitschrift der Gesellschaft Deutscher Chemiker

Supporting Information

Pseudo Natural Products—Chemical Evolution of Natural Product Structure

*George Karageorgis, Daniel J. Foley, Luca Laraia, Susanne Brakmann, and Herbert Waldmann**

anie_202016575_sm_miscellaneous_information.pdf

Author Contributions

G.K. performed the cheminformatic analyses. All authors wrote and commented on the manuscript.

Molecular properties were calculated using RDKit (http://www.rdkit.org) running in Python 3.7.

Plots were generated using Matplotlib.[1]

Molecular properties of individual pseudo-NP classes used for the cheminformatic analyses:

Pyranofuranopyridones[2]

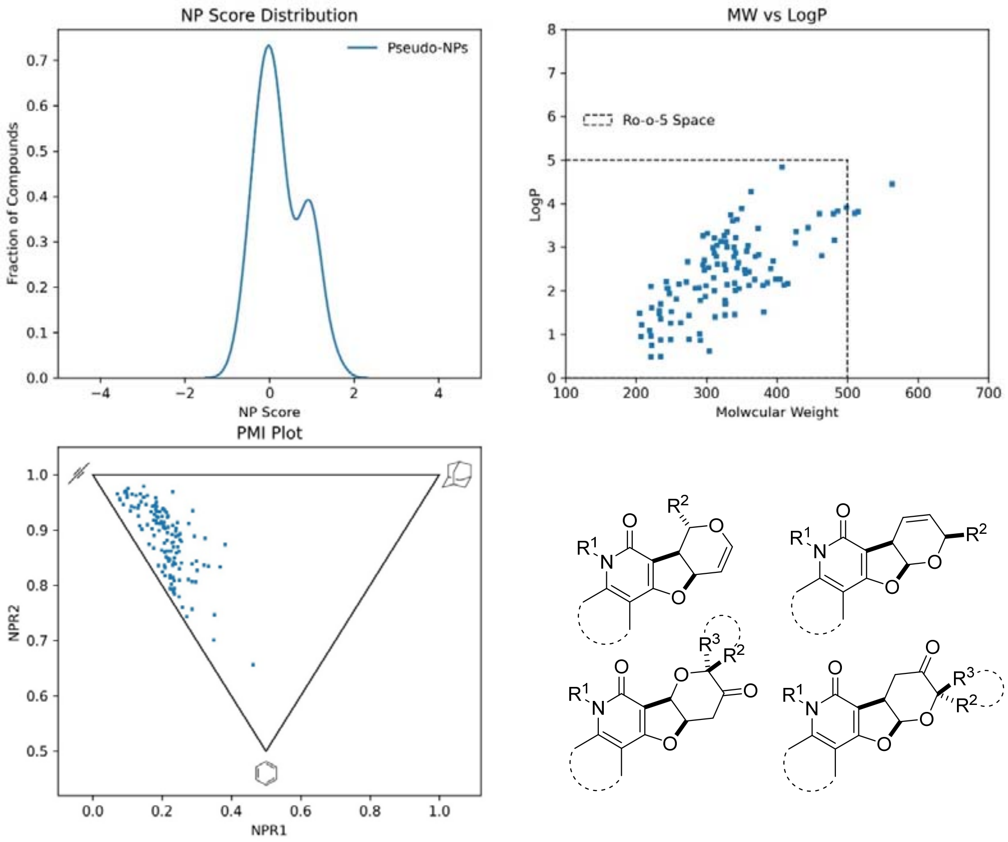

Figure S1: Molecular properties of pyranofuranopyridone pseudo-NPs. Top left: NP Score distribution. Top right: Molecular Weight against LogP. Dashed box denotes “Rule-of-five” space. Bottom left: PMI plot[3] of pyranofuranopyridone scaffold derivatives. Bottom right: Pyranofuranopyridone scaffolds structures.

Indomorphans[4]

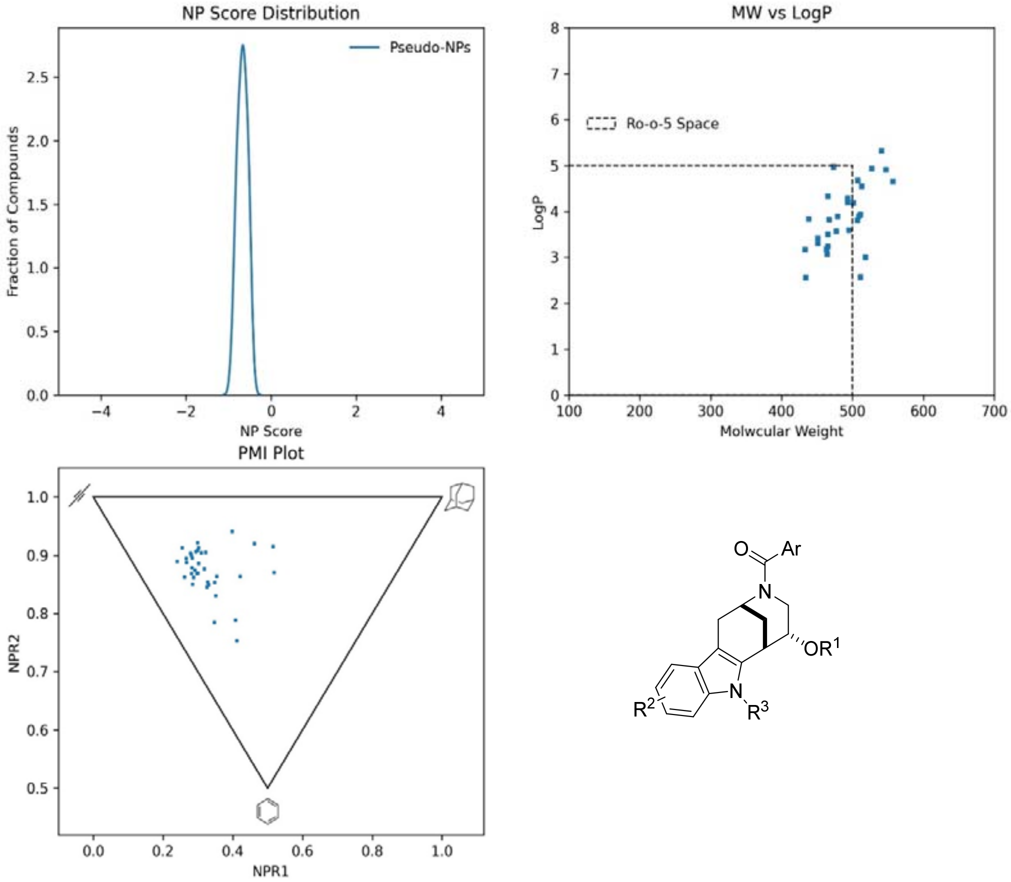

Figure S2: Molecular properties of indomorphan pseudo-NPs. Top left: NP Score distribution. Top right: Molecular Weight against LogP. Dashed box denotes "Rule-of-five" space. Bottom left: PMI plot[3] of indomorphan scaffold derivatives. Bottom right: Indomorphan scaffold structures.

Pyrroquinolines[5]

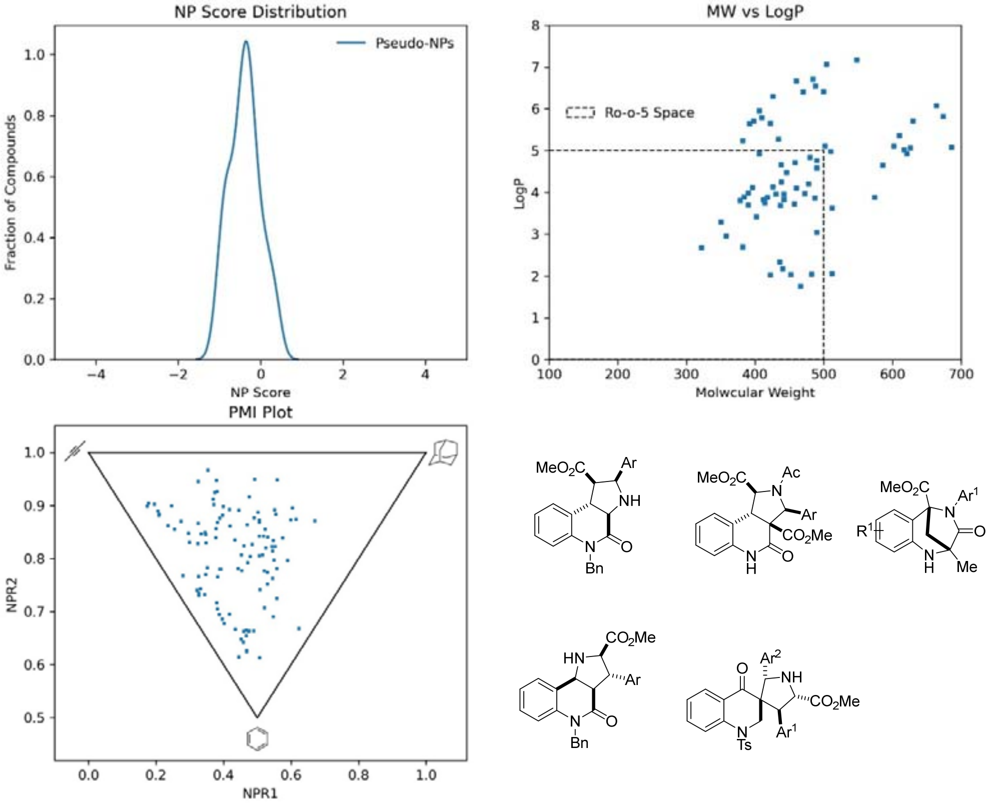

Figure S3: Molecular properties of pyrroquinoline pseudo-NPs. Top left: NP Score distribution. Top right: Molecular Weight against LogP. Dashed box denotes “Rule-of-five” space. Bottom left: PMI plot[3] of pyrroquinoline scaffold derivatives. Bottom right: pyrroquinoline scaffold structures.

Pseudo-NPs based on cinchona alkaloids[6,7]

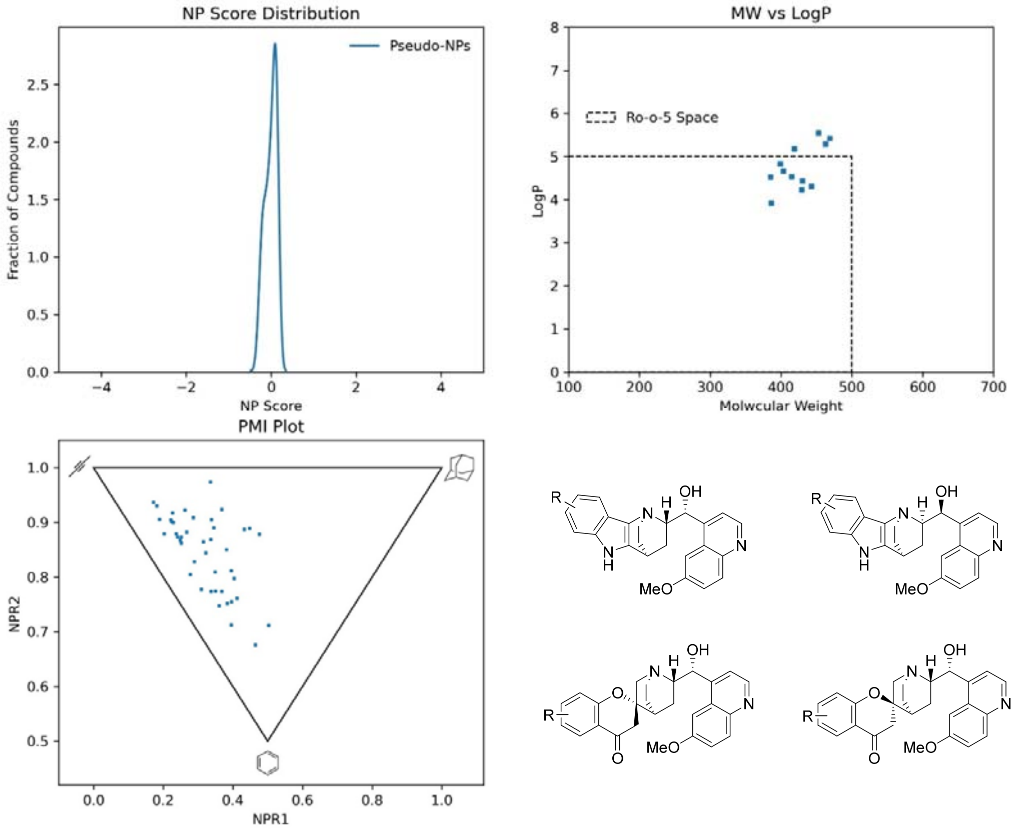

Figure S4: Molecular properties of cinchona pseudo-NPs. Top left: NP Score distribution. Top right: Molecular Weight against LogP. Dashed box denotes “Rule-of-five” space. Bottom left: PMI plot[3] of cinchona pseudo-NP derivatives. Bottom right: cinchona pseudo-NP scaffold structures.

Pseudo-NPs based on sinomenine, and griseofulvin[7]

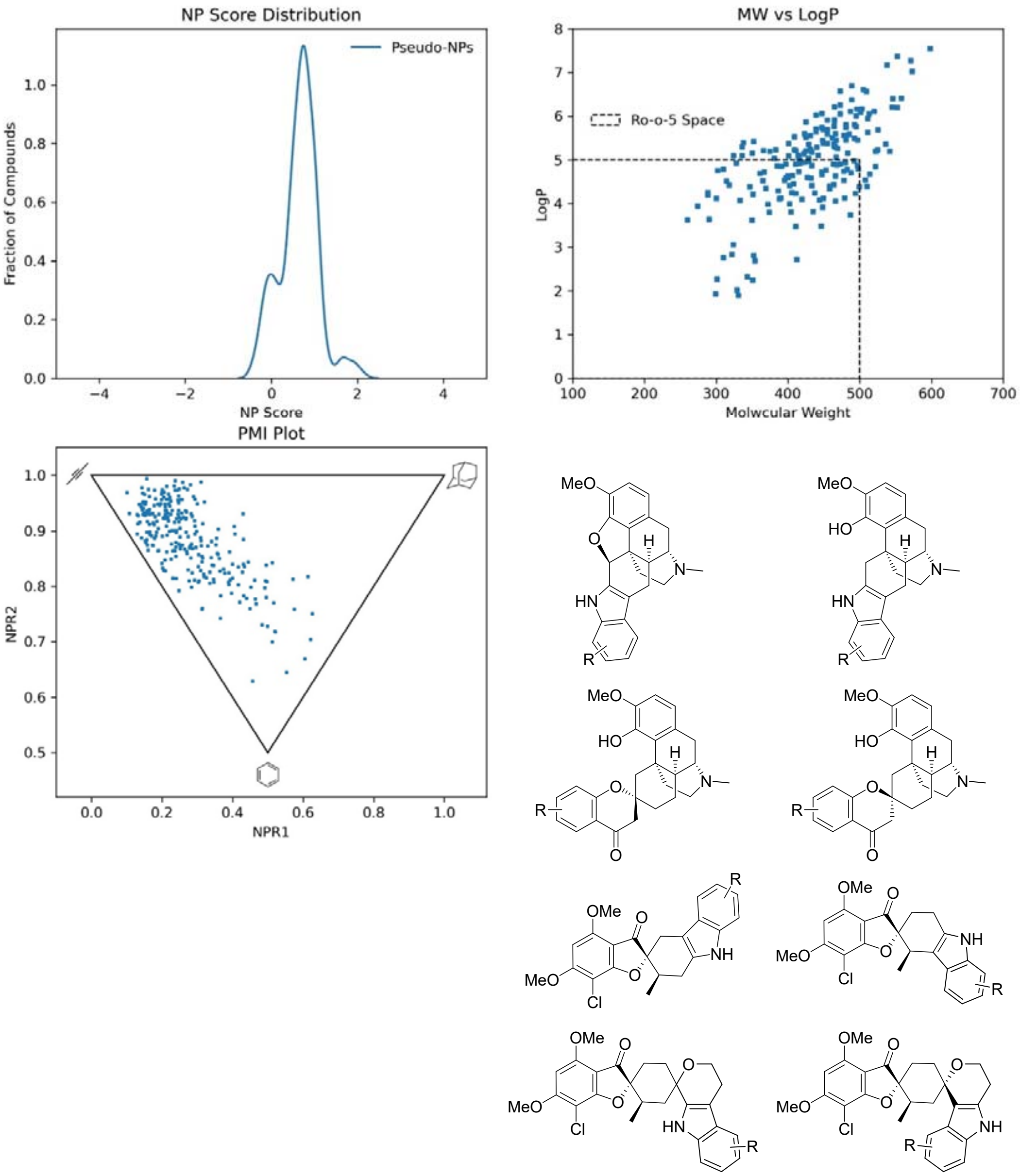

Figure S5: Molecular properties of sinomenine, and griseofulvin pseudo-NPs. Top left: NP Score distribution. Top right: Molecular Weight against LogP. Dashed box denotes “Rule-of-five” space. Bottom left: PMI plot[3] of sinomenine, and griseofulvin pseudo-NP derivatives. Bottom right: sineomenine, and griseofulvin pseudo-NP scaffold structures.

Chromopynones[8]

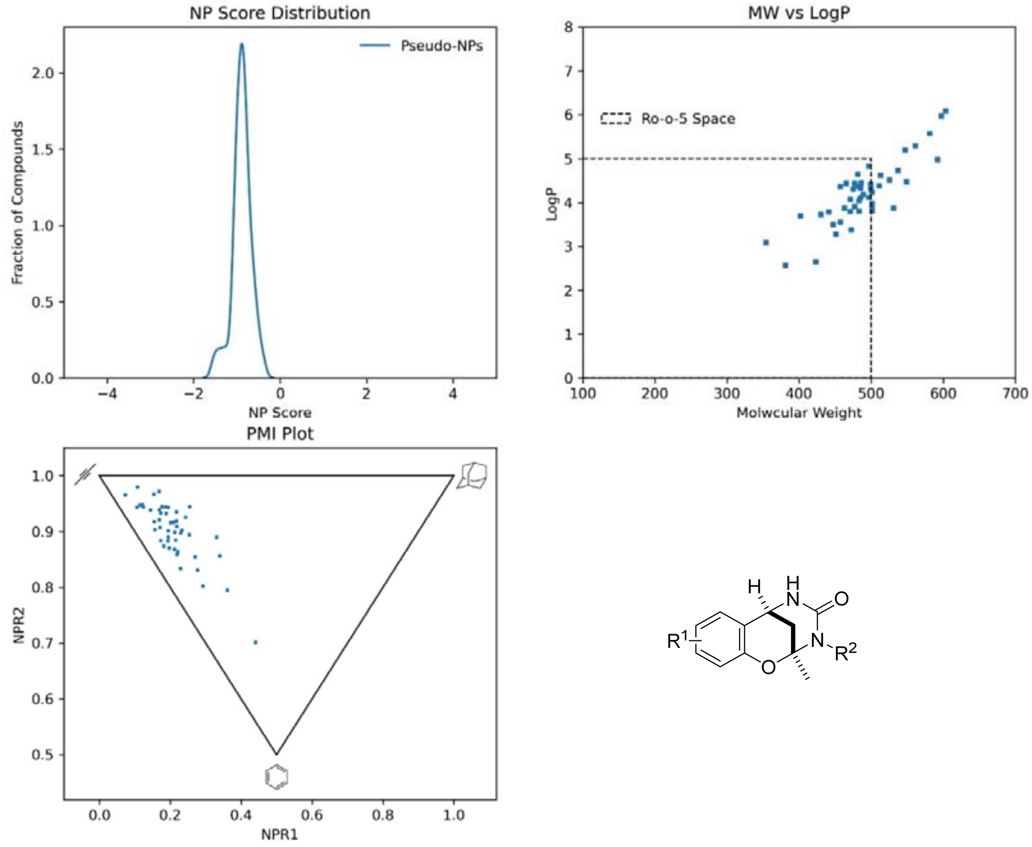

Figure S6: Molecular properties of chromopynone pseudo-NPs. Top left: NP Score distribution. Top right: Molecular Weight against LogP. Dashed box denotes “Rule-of-five” space. Bottom left: PMI plot[3] of chromopynone scaffold derivatives. Bottom right: chromopynone scaffold structure.

Table S1: Molecular properties of combined pseudo-NP libraries, averages and ranges.

	Mean	Range	Lit. Guidelines
Molecular Weight	426	205 - 686	150-500
LogP	4.26	0.47 - 7.61	0-5
Hydrogen-bond Acceptors	5	1 - 10	< 10
Hydrogen-bond Donors	1	0 - 3	< 5
Total Polar Surface Area ($Å^2$)	64	8 - 136	< 140
Fraction of sp^3 C atoms	0.36	0.08 - 0.72	0.3-0.6

Literature guidelines referred in literature[9–13]

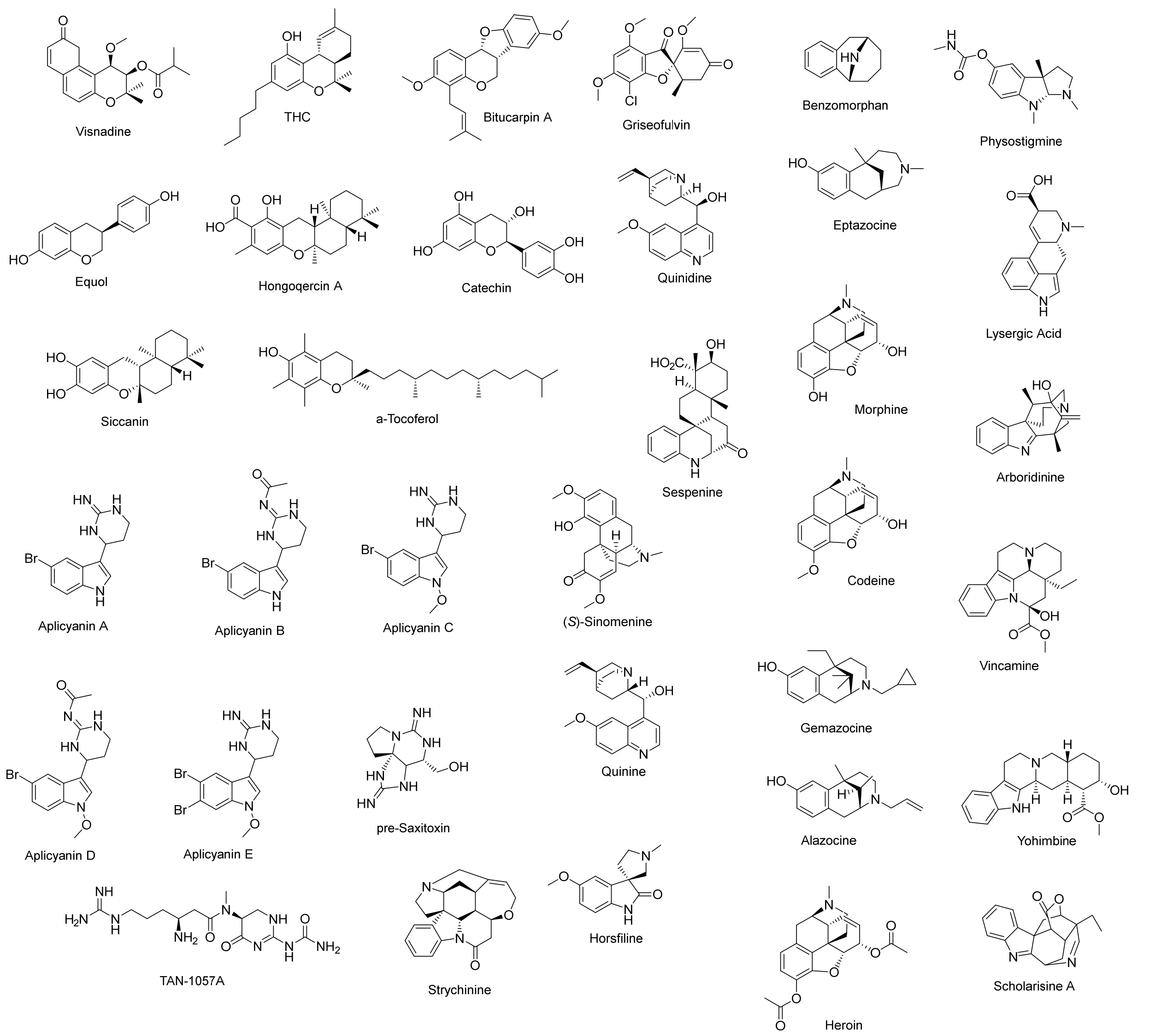

Figure S7: Structures and names for NPs and bioactive compounds used to prepare the PMI plot II in Figure 3.

References:

[1] J. D. Hunter, *Comput. Sci. Eng.* **2007**, *9*, 90–95.

[2] A. Christoforow, J. Wilke, A. Binici, A. Pahl, C. Ostermann, S. Sievers, H. Waldmann, *Angew. Chem. Int. Ed.* **2019**, *58*, 14715–14723.

[3] W. H. B. Sauer, M. K. Schwarz, *J. Chem. Inf. Comput. Sci.* **2003**, *43*, 987–1003.

[4] J. Ceballos, M. Schwalfenberg, G. Karageorgis, E. S. Reckzeh, S. Sievers, C. Ostermann, A. Pahl, M. Sellstedt, J. Nowacki, M. A. Carnero Corrales, et al., *Angew. Chem. Int. Ed.* **2019**, *58*, 17016–17025.

[5] H. Waldmann, J. Liu, G. S. Cremosnik, F. Otte, A. Pahl, S. Sievers, C. Strohmann, *Angew. Chem. Int. Ed.* **2020**, anie.202013731.

[6] D. J. Foley, S. Zinken, D. Corkery, L. Laraia, A. Pahl, Y.-W. Wu, H. Waldmann, *Angew. Chem. Int. Ed.* **2020**, *59*, 12470–12476.

[7] M. Grigalunas, A. Burhop, S. Zinken, A. Pahl, S. Sievers, D. J. Foley, A. P. Antonchick, H. Waldmann, *Nat. Commun, Revis.* **n.d.**

[8] G. Karageorgis, E. S. Reckzeh, J. Ceballos, M. Schwalfenberg, S. Sievers, C. Ostermann, A. Pahl, S. Ziegler, H. Waldmann, *Nat. Chem.* **2018**, *10*, 1103–1111.

[9] D. B. Kell, S. G. Oliver, *Front. Pharmacol.* **2014**, *5*, 1–32.

[10] C. A. Lipinski, F. Lombardo, B. W. Dominy, P. J. Feeney, *Adv. Drug Deliv. Rev.* **1997**, *23*, 3–25.

[11] D. F. Veber, S. R. Johnson, H.-Y. Cheng, B. R. Smith, K. W. Ward, K. D. Kopple, *J. Med. Chem.* **2002**, *45*, 2615–2623.

[12] F. Lovering, *Medchemcomm* **2013**, *4*, 515–519.

[13] F. Lovering, J. Bikker, C. Humblet, *J. Med. Chem.* **2009**, *52*, 6752–6756.
